# A systematic review of immune checkpoint inhibitors in endometrial cancer

**DOI:** 10.3389/fonc.2026.1776831

**Published:** 2026-06-08

**Authors:** Na Zhu, Chaihong Zhang

**Affiliations:** 1 Clinical Trial Institution Office, Shaanxi Provincial People’s Hospital, Xi’an, Shaanxi, China; 2 Core Research Laboratory, Second Affiliated Hospital, Xi’an Jiaotong University, Xi’an, Shaanxi, China; 3 Department of Obstetrics and Gynecology, Shaanxi Provincial People’s Hospital, Xi’an, Shaanxi, China

**Keywords:** Bibliometrix, CiteSpace, endometrial cancer, immune checkpoint inhibitors, VOSviewer

## Abstract

**Background:**

Endometrial cancer represents one of the most prevalent gynecological malignancies. This study aims to systematically analyze the knowledge frameworks, key contributors, research hotspots, emerging trending topics, and future directions in the field of immune checkpoint inhibitors for endometrial cancer by applying bibliometric methodologies.

**Methods:**

A thorough and methodical search of the Web of Science Core Collection (WoSCC) database and the PubMed database was performed to identify publications related to immune checkpoint inhibitors in endometrial cancer from January 1, 2015, to December 31, 2024. Furthermore, bibliometric analysis and knowledge map visualization were conducted utilizing VOSviewer, Biblioshiny, and CiteSpace.

**Results:**

For the WoSCC database, this research involved an analysis of 829 publications disseminated across 296 journals, authored by 5,145 researchers affiliated with 1,535 institutions from 63 different countries. For the PubMed database, this research involved an analysis of 279 publications disseminated across 120 journals, authored by 1,611 researchers affiliated with 974 institutions from 46 different countries. The leading three contributors to publication volume were United States, China, and Italy. Importantly, the highest level of international collaboration has been recorded between United States and England. Gynecologic Oncology, Cancers, Frontiers in Immunology, Frontiers in Oncology, and the Journal of Clinical Oncology are acknowledged as the five most influential journals regarding immune checkpoint inhibitors for endometrial cancer. Makker Vicky emerged as the most influential author, while Memorial Sloan Kettering Cancer Center was identified as the most influential institution. Current research hotspots regarding immune checkpoint inhibitors in the treatment of endometrial cancer include microsatellite instability, mismatch repair deficiency, PD-1, PD-L1, chemotherapy, pembrolizumab, lenvatinib, and dostarlimab. Additionally, emerging trending topics in this field encompass pembrolizumab, lenvatinib, dostarlimab, immune checkpoint inhibitors, and mismatch repair deficiency.

**Conclusion:**

An in-depth exploration of the molecular mechanisms of immune checkpoint inhibitors for endometrial cancer is crucial for overcoming the current clinical challenges associated with immunotherapy. This study systematically analyzes the current state of research in this field, focusing on research hotspots, emerging trends, and future development directions. Additionally, it comprehensively evaluates the most influential literature, core journals, authoritative scholars, leading research institutions, and major contributing countries in this field.

## 1 Introduction

Endometrial cancer represents one of the most prevalent gynecological malignancies, and its incidence has been increasing in recent decades (1, 2). In 1983, Bokhman proposed a classification system for endometrial cancers based on two distinct pathogenetic types. The first type exhibits high sensitivity to progestogens and is associated with a favorable prognosis. In contrast, the second type shows reduced sensitivity to progestogens and is linked to a doubtful prognosis (3). However, the pathogenetic classification system is undergoing a transitional phase and is being replaced by the molecular typing system developed by The Cancer Genome Atlas (TCGA) (4, 5). The TCGA classified endometrial cancers into four distinct types: DNA Polymerase Epsilon (POLE) ultramutated, microsatellite instability hypermutated, copy-number low, and copy-number high (4). The prognosis associated with the POLE ultramutated subtype is considered the most favorable, in contrast to the copy-number high subtype, which is associated with the least favorable prognosis (5). The TCGA molecular typing system not only provides a crucial basis for assessing the prognosis of endometrial cancer but also establishes a foundation for the precise application of immunotherapy (6). In recent years, with the clinical application of TCGA molecular typing, the treatment model of endometrial cancer is undergoing a revolutionary transformation from traditional histopathological typing to precise treatment guided by molecular characteristics (7).

Traditionally, the treatment for endometrial cancer has included surgical intervention, radiotherapy, chemotherapy, and hormonal therapy (5, 8, 9). While early-stage patients can achieve better therapeutic benefits from surgical intervention and adjuvant radiotherapy/chemotherapy, the treatment of advanced or recurrent cases presents significant challenges (10, 11). The efficacy of traditional treatment methods for these advanced or recurrent cases is limited (10), highlighting the urgent need to explore more effective treatment strategies. In recent years, tumor immunotherapy, especially the rapid advancement of immune checkpoint inhibitors, has led to significant breakthroughs in the treatment of various malignant tumors (12, 13). Endometrial cancer is acknowledged as an immunogenic tumor (11), characterized by a high proportion of tumor mutational burden (TMB), mismatch repair deficiency (dMMR), or microsatellite instability-high (MSI-H) (11, 14, 15). It shows significant sensitivity to immunotherapy (11, 16). Immune checkpoint inhibitors have emerged as novel therapeutic alternatives for patients diagnosed with endometrial cancer, offering renewed hope for their treatment (17, 18).

Medical bibliometric analyses quantitatively assess the research contributions and academic influences of authors, institutions, countries, and journals to identify research hotspots in the field, track emerging trending topics in the discipline, and reveal important trends in disciplinary innovation (19). These analyses provide essential data support and a scientific foundation for strategic planning in scientific research, the formulation of healthcare policies, and the optimization of clinical practice guidelines (20). Nevertheless, there remains a notable deficiency in bibliometric analyses pertaining to publications focused on immune checkpoint inhibitors in the context of endometrial cancer. We did not retrieve any publications in the Web of Science or PubMed databases that focused on the bibliometric analysis of immune checkpoint inhibitors in endometrial cancer. This study utilizes VOSviewer software (21), the Biblioshiny online platform based on the Bibliometrix package (https://www.bibliometrix.org) (22), as well as CiteSpace software (23), to perform a bibliometric analysis of documents related to immune checkpoint inhibitors for endometrial cancer from 2015 to 2024. The aim is to develop knowledge maps that delineate significant contributors, research hotspots, and emerging trending topics, in addition to exploring trends in innovation and potential avenues for development.

## 2 Materials and methods

### 2.1 Data source and retrieval strategy

**Figure 1:**
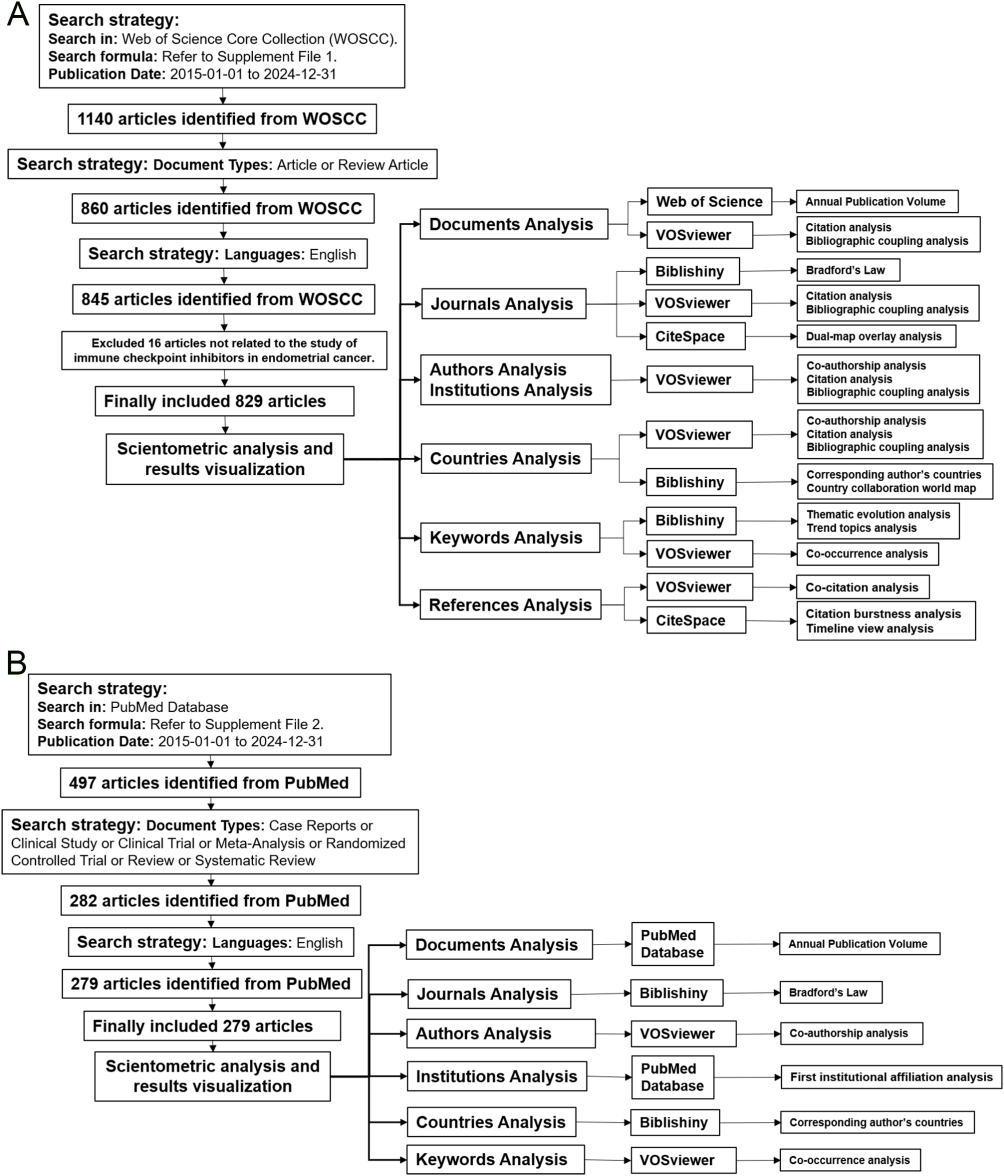
The flowchart illustrates the methodology employed for the screening of documents and the conduct of bibliometric analysis. **(A)** The data are obtained from the Web of Science database. **(B)** The data are obtained from the PubMed database.

On March 7, 2025, a thorough and methodical search of the Web of Science Core Collection (WoSCC) database (https://webofscience.clarivate.cn/wos/woscc) was performed to identify publications related to immune checkpoint inhibitors in endometrial cancer, covering the period from January 1, 2015, to December 31, 2024. Similarly, on December 14, 2025, a thorough search of the PubMed database (https://pubmed.ncbi.nlm.nih.gov/) was performed for the same topic and period The criteria for inclusion in this study were delineated as follows (1): studies pertaining to immune checkpoint inhibitors in endometrial cancer; (2) sources limited to the WoSCC or PubMed; (3) a restriction to document types that include only articles and review articles; (4) publications available in the English language; and (5) a temporal scope for the search that spans from January 1, 2015, to December 31, 2024. In contrast, the exclusion criteria encompassed the following: (1) studies that do not pertain to immune checkpoint inhibitors in endometrial cancer; (2) publications that are not written in English; (3) document types that are not classified as either articles or review articles; and (4) duplicate publications. The search formulas are detailed in **Supplementary Files 1**, **2**. The search strategy is detailed in **Figure 1**. A total of 829 and 279 articles were identified from WOSCC and PubMed, respectively. The complete records and cited references for each publication were then extracted and saved as plain text files. The procedures for data collection and screening were carried out by two researchers. **Figure 1** shows the flowchart for literature retrieval and bibliometric analysis. According to the requirements of Frontiers’ journals, PubMed exists as a separate, non-overlapping validation database, which is used to verify the consistency of the analysis results from the Web of Science database. We conducted a search of the Web of Science database on March 7, 2025, and a search of the PubMed database on December 14, 2025. The temporal gap between the two searches could also introduce database update bias. In this study, even when there is a temporal gap, the consistency of the analysis results from the Web of Science and PubMed data remains strong.

### 2.2 Bibliometric analyses and visualization

For the purpose of analyzing the annual publication volume and the cumulative annual publications sourced from WoSCC or PubMed, and conducting linear regression analysis, Microsoft Office Excel 2019 (Microsoft, Redmond, WA, USA) was employed. Additionally, Microsoft Office Excel 2019 was used to analyze the first institutional affiliations for the 279 articles sourced from PubMed. Finally, bibliometric analysis and visualization were conducted utilizing VOSviewer (21), Bibliometrix (22), and CiteSpace (23).

#### 2.2.1 VOSviewer analyses

##### 2.2.1.1 Web of science core collection database

VOSviewer (version 1.6.20) is a free software application that was developed for constructing and displaying bibliometric networks and visualized maps (21). In the present study, VOSviewer was applied to perform co-authorship analyses of authors, institutions, and countries; to conduct co-occurrence analyses of author keywords; to execute citation or bibliographic coupling analyses of documents, journals, authors, institutions, and countries; and to carry out co-citation analyses of references. In the generated network visualization maps, the dimensions of the nodes correspond to the quantity or frequency of relevant elements, while the thickness of the lines connecting the nodes reflects the strength of interactions. Furthermore, distinct colors are utilized to represent different clusters.

VOSviewer was applied to visualize co-authorship, citation, or bibliographic coupling networks among authors, institutions, or countries. When the minimum number of documents for an author, institution, or country was set at one, and the minimum number of citations for the same categories was set at zero, we identified 5,145 authors, 1,535 institutions, and 63 countries. For each identified entity, we calculated the total strength of co-authorship, citation, or bibliographic coupling links with other entities. We filtered the top 10 authors based on the “Number of documents” to select those who have published the most articles. Since several authors have the same number of published articles, a total of 13 authors were selected when the “Number of documents” was set to greater than or equal to eight. Then, we sorted these 13 authors based on the “Citations” and chose the top 10 authors when the “Citations” was set to greater than or equal to 140. We filtered the top 10 institutions based on the “Number of documents” to select those that have published the most articles. A total of 10 institutions were selected when the “Number of documents” was set to greater than or equal to 13. The citations among the 10 institutions were greater than or equal to 700. We filtered the top 10 countries based on the “Number of documents” to select those that have published the most articles. A total of 10 countries were selected when the “Number of documents” was set to greater than or equal to 30. The citations among the 10 countries were greater than or equal to 2,000. To sum up, the criteria for the networks among authors were specified as follows: the minimum number of documents for an author was set at eight, and the minimum number of citations for an author was set at 140. The criteria for the networks among institutions were specified as follows: the minimum number of documents for an institution was set at 13, and the minimum number of citations for an institution was set at 700. The criteria for the networks among countries were specified as follows: the minimum number of documents for a country was set at 30, and the minimum number of citations for a country was set at 2,000.

VOSviewer was also utilized to visualize citation or bibliographic coupling networks among documents or journals. When the minimum number of citations for a document was set at zero, we identified 829 documents. Both global and local citation counts were determined for each document, leading to the identification of the top ten documents with the highest global or local citations. For journals, the minimum number of documents was set at one, and the minimum number of citations was set at zero, resulting in the identification of 296 journals. For each identified journal, we calculated the total strength of citation or bibliographic coupling links with other journals. For core journals, VOSviewer was utilized to visualize citation or bibliographic coupling networks.

Additionally, VOSviewer was employed to visualize co-occurrence networks of author keywords. When the minimum number of occurrences of a keyword was set at one, we identified 1,123 keywords. For each keyword, the frequency of occurrence was assessed, and the total strength of co-occurrence links with other keywords was also calculated.

Finally, VOSviewer was utilized to visualize a co-citation network for cited references. When the minimum number of citations for a cited reference was set at one, we identified 24,043 cited references. The total strength of co-citation links with other references was calculated for each identified reference. For each reference, the local citations were assessed, culminating in the selection of the top ten references with the highest local citations.

##### 2.2.1.2 PubMed database

In the present study, VOSviewer was applied to perform co-authorship analyses of authors and to conduct co-occurrence analyses of author keywords.

VOSviewer was applied to visualize the co-authorship network among authors. When the minimum number of documents for an author was set at one, we identified 1,611 authors. For each identified author, we calculated the total strength of co-authorship links with other authors. The criteria for the network among authors were specified as follows: the minimum number of documents for an author was set at eight. In the generated network visualization map, the dimensions of the nodes correspond to the quantity or frequency of relevant elements, while the thickness of the lines connecting the nodes reflects the strength of interactions.

Additionally, VOSviewer was employed to visualize co-occurrence networks of author keywords. When the minimum number of occurrences of a keyword was set at one, we identified 423 keywords. For each keyword, the frequency of occurrence was assessed.

#### 2.2.2 Bibliometrix analyses

##### 2.2.2.1 Web of science core collection database

Bibliometrix (version 4.3.2) is a software package developed in R (version 4.4.2) (https://www.r-project.org/) that features a Biblioshiny web interface, facilitating extensive science mapping analysis (22). In the present study, Bibliometrix was employed to conduct core journal analysis, corresponding authors’ countries analysis, country collaboration map analysis, thematic evolution analysis, and trend topics analysis.

**Figure 2:**
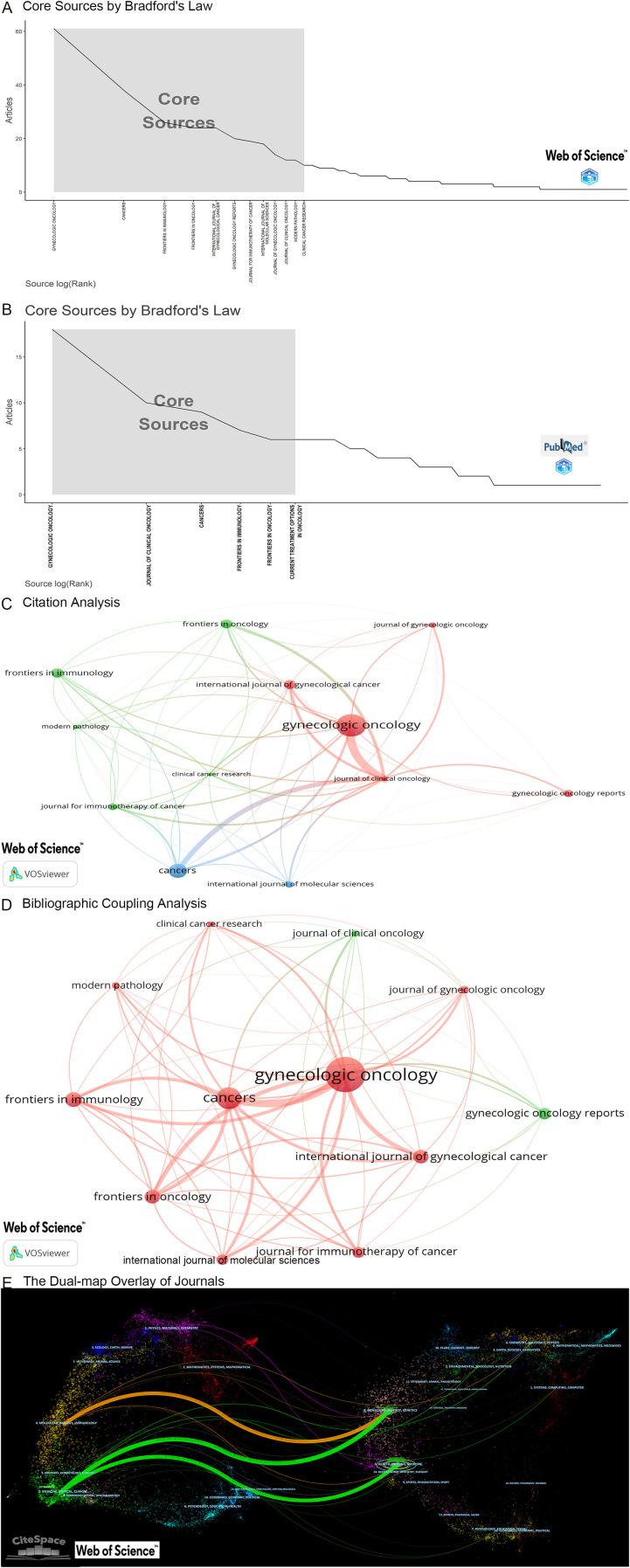
Journal analyses. **(A, B)**. The screening of core journals in alignment with Bradford’s Law. **(A)** The data are obtained from the Web of Science database. **(B)** The data are obtained from the PubMed database. **(C–E)**. The data are obtained from the Web of Science database. **(C, D)**. The two networks depict the analyses of citation and bibliographic coupling for the twelve core journals, respectively. The dimensions of each node correspond to the quantity of documents, the thickness of the lines reflects the intensity of interactions, and the colors signify distinct clusters. **(E)** The dual-map overlay analysis of journals. The map illustrates the distribution of journal disciplines between the citing field and the cited field. The citation curve depicts the transfer of knowledge from the citing field to the cited field, with distinct colors denoting various academic disciplines.

Bibliometrix was utilized to conduct a core journal analysis based on Bradford’s Law. The journals in the shaded areas of **Figure 2A** are all core journals. Bibliometrix was also applied to perform a corresponding authors’ countries analysis with a restriction to ten countries to quantify the number of single-country publications (SCP) and multiple-country publications (MCP). Additionally, Bibliometrix was employed to facilitate the visualization of a country’s collaboration world map, with the minimum edges set at 20 and the edge size set at five. Furthermore, Bibliometrix was utilized to execute thematic evolution analyses using the Walktrap clustering algorithm in the field of author keywords from 2015 to 2024, with the number of keywords set at 200 and the minimum cluster frequency per thousand documents set at 30. Lastly, Bibliometrix was applied to facilitate a trend topics analysis in the field of author keywords from 2015 to 2024, with the minimum keyword frequency set at 30 and the number of keywords per year set at 10.

##### 2.2.2.2 PubMed database

In the present study, Bibliometrix was utilized to conduct a core journal analysis based on Bradford’s Law. The journals in the shaded areas of **Figure 2B** are all core journals. Bibliometrix was also applied to perform a corresponding authors’ countries analysis, restricted to ten countries, to quantify the number of SCP and MCP.

#### 2.2.3 CiteSpace analyses of the data sourced from the web of science core collection

CiteSpace (version 6.1.6) serves as a tool for bibliometric analysis and visualization (23). In the present study, CiteSpace was employed to perform analyses on journals and cited references.

CiteSpace was utilized to conduct a dual-map overlay analysis on journals using the method of standardization of z-scores, with the label top N journals set at ten, the α set at one, the source circle size set at ten, the target circle size set at five, and the snap to centroids set at zero. The dual map consists of the citing journals map and the cited journals map, respectively. Furthermore, CiteSpace was applied to perform a burstness analysis on cited references, with the number of states set at two, the minimum duration set at two, and the burst items found set at zero. Lastly, CiteSpace was employed to execute a cluster and timeline analysis on cited references. The timeline view map labeled clusters with indexing terms and showed cluster labels by the log-likelihood ratio method.

## 3 Results

### 3.1 Document analyses

#### 3.1.1 Document analyses based on web of science core collection database

##### 3.1.1.1 Annual publications on immune checkpoint inhibitors in endometrial cancer

**Figure 3:**
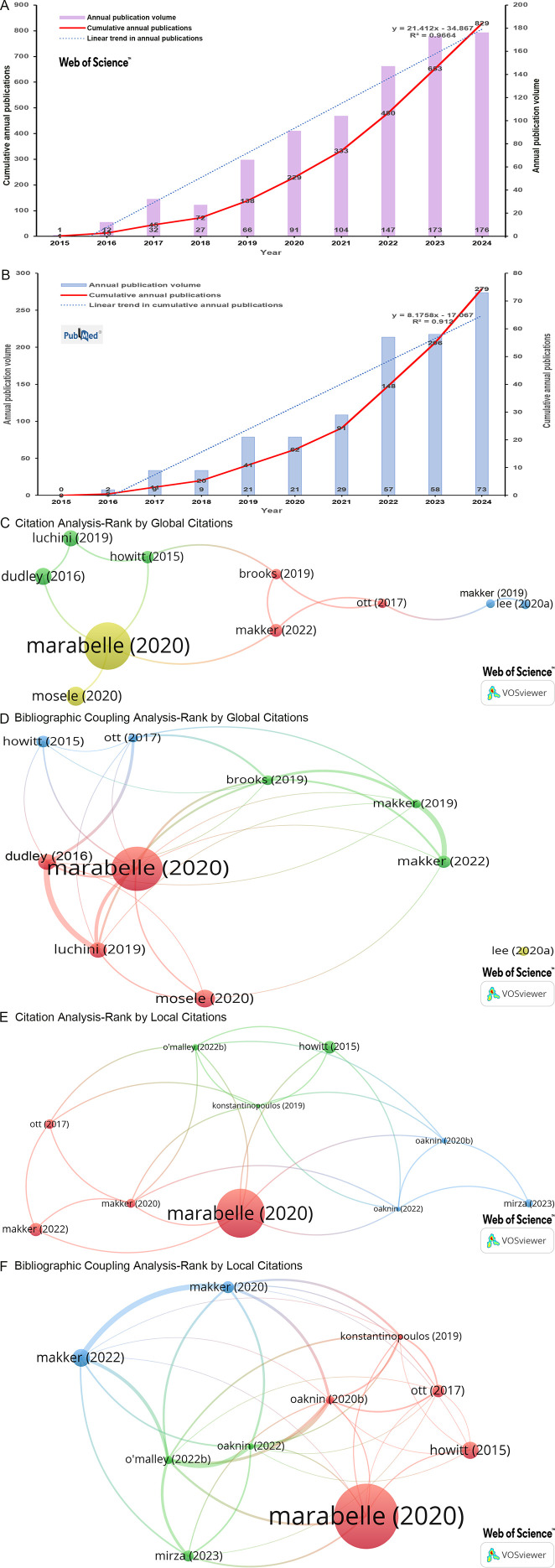
Document analyses. **(A, B)**. The annual and cumulative growth in research publications related to immune checkpoint inhibitors in endometrial cancer is outlined. **(A)** The data are obtained from the Web of Science database. The annual publication volume is illustrated by the pink column, which denotes the number of publications generated each year. The cumulative annual publications are represented by the red line, reflecting the aggregate number of publications accumulated up to each corresponding year. The blue dotted line depicts the linear trend in annual publications, as determined by a linear regression analysis. **(B)** The data are obtained from the PubMed database. The annual publication volume is illustrated by the blue column, which denotes the number of publications generated each year. The cumulative annual publications are represented by the red line, reflecting the aggregate number of publications accumulated up to each corresponding year. The blue dotted line depicts the linear trend in annual publications, as determined by a linear regression analysis. **(C–F)**. The data are obtained from the Web of Science database. The dimensions of the nodes are indicative of citation frequency, whereas the thickness of the lines reflects the intensity of interactions, with varying colors denoting distinct clusters. **(C, D)**. The two networks illustrate the citation and bibliographic coupling analyses of the ten most frequently cited articles on a global scale, respectively. **(E, F)**. The two networks depict the analyses of citation and bibliographic coupling pertaining to the ten most frequently cited articles on a local scale, respectively.

A total of 829 publications concerning immune checkpoint inhibitors in endometrial cancer were collected, covering the period from January 1, 2015, to December 31, 2024. Only one article was published in 2015. Since 2019, the number of published articles has exceeded 50 annually. By 2021, this figure surpassed 100, ultimately reaching a total of 176 articles by 2024. With the exception of a brief decline in 2018, the overall trend in the number of published articles has demonstrated a consistent upward trajectory. Notably, from 2021 to 2024, there was a substantial increase in publication output, with figures rising from 104 to 176 (**Figure 3A**). The blue dotted line depicted in **Figure 3A** illustrates a linear regression curve (R² = 0.9664), which accurately reflects the annual growth trajectory of publications related to immune checkpoint inhibitors in endometrial cancer.

##### 3.1.1.2 Global citations analysis

Global citations and local citations are two important indicators for assessing the impact of scholarly literature. Global citations refer to the total number of times a document is cited by other documents within the Web of Science database. **Table 1** displays the top ten most globally cited articles, among which four have more than 600 citations, with Marabelle (2020) being the most globally cited, followed by Mosele (2020), Dudley (2016), and Luchini (2019).

**Table 1:** Top ten most cited articles on immune checkpoint inhibitor studies in endometrial cancer.

Top ten most globally cited articles (WOSCC)						Top ten most locally cited articles (WOSCC)					
First author (date)	GC	LC	LC/GC	NGC	NLC	First author (date)	GC	LC	LC/GC	NGC	NLC
marabelle (2020)	1902	179	9.41	27.65	23.11	ott (2017)	370	181	48.92	5.21	14.52
mosele (2020)	769	0	0.00	11.18	0.00	marabelle (2020)	1902	179	9.41	27.65	23.11
dudley (2016)	679	51	7.51	3.94	2.30	makker (2022)	525	154	29.33	24.86	33.32
luchini (2019)	645	44	6.82	10.44	4.81	howitt (2015)	502	145	28.88	1.00	1.00
makker (2022)	525	154	29.33	24.86	33.32	makker (2020)	356	126	35.39	5.18	16.27
howitt (2015)	502	145	28.88	1.00	1.00	oaknin (2020b)	243	103	42.39	3.53	13.30
brooks (2019)	426	55	12.91	6.89	6.02	konstantinopoulos (2019)	159	89	55.97	2.57	9.74
lee (2020a)	377	4	1.06	5.48	0.52	o’malley (2022b)	251	89	35.46	11.88	19.26
ott (2017)	370	181	48.92	5.21	14.52	mirza (2023)	326	81	24.85	30.13	37.43
makker (2019)	367	77	20.98	5.94	8.42	oaknin (2022)	192	79	41.15	9.09	17.09

GC, Global Citations; LC, Local Citations; LC/GC, LC/GC Ratio (%); NGC, Normalized Global Citations; NLC, Normalized Local Citations.

The citation analysis counts the reciprocal citation frequency among the top ten most globally cited articles. A citation network was visualized based on the ten articles (**Figure 3C**). Marabelle (2020) was the most globally cited article and was recognized as the core of this network, demonstrating mutual citation relationships with the other four articles (links = 4) (**Figure 3C**).

The bibliographic coupling analysis counts the citation frequency of shared references across the ten articles. A bibliographic coupling network was visualized for the ten articles (**Figure 3D**). Marabelle (2020) was the most globally cited article and served as the core of this network, sharing the same references with the other eight articles (links = 18) (**Figure 3D**).

##### 3.1.1.3 Local citations analysis

Local citations refer to the total number of times an article is cited by 828 other articles. **Table 1** shows the top ten most locally cited articles, among which three have more than 150 citations, with Ott (2017) emerging as the most locally cited, followed by Marabelle (2020) and Makker (2022).

A citation network was visualized based on the top ten most locally cited articles (**Figure 3E**). Ott (2017), Marabelle (2020), and Makker (2022) were the three most locally cited articles. Compared with the other two articles, Marabelle (2020) served as more central to the network, demonstrating citation relationships with the other six articles (links = 6) (**Figure 3E**).

Furthermore, a bibliographic coupling network was visualized for the top ten most locally cited articles (**Figure 3F**). Ott (2017), Marabelle (2020), and Makker (2022) were the three most locally cited articles. Ott (2017) shared the same references with the other eight articles (links = 13). Marabelle (2020) shared the same references with the other eight articles (links = 17). Makker (2022) shared the same references with the other seven articles (links = 25) (**Figure 3F**).

#### 3.1.2 Document analyses based on PubMed database

A total of 279 publications concerning immune checkpoint inhibitors in endometrial cancer were collected, covering the period from January 1, 2015, to December 31, 2024 (**Figure 3B**). The annual and cumulative growth trends in the number of articles published from 2018 to 2024 in the WoSCC and PubMed databases are highly consistent (**Figure 3B**).

To summarize, Marabelle (2020), Mosele (2020), Dudley (2016), Luchini (2019), Ott (2017), and Makker (2022) are the six most important articles concerning immune checkpoint inhibitors in endometrial cancer. Marabelle (2020), published in the Journal of Clinical Oncology (Impact Factor = 41.9, Q1), lists the corresponding author’s affiliated institution as Memorial Sloan Kettering Cancer Center in United States. Mosele (2020), published in Annals of Oncology (Impact Factor = 65.4, Q1), lists the corresponding author’s affiliated institution as Gustave Roussy Institute in France. Dudley (2016), published in Clinical Cancer Research (Impact Factor = 10.2, Q1), lists the corresponding author’s affiliated institution as Johns Hopkins University in United States. Luchini (2019), published in Annals of Oncology (Impact Factor = 65.4, Q1), lists the corresponding author’s affiliated institution as Gustave Roussy Institute in France. Ott (2017), published in the Journal of Clinical Oncology (Impact Factor = 41.9, Q1), lists the corresponding author’s affiliated institution as Dana-Farber Cancer Institute in United States. Makker (2022), published in the New England Journal of Medicine (Impact Factor = 78.5, Q1), lists the corresponding author’s affiliated institution as Memorial Sloan Kettering Cancer Center in United States.

### 3.2 Journal analyses

#### 3.2.1 Journal analyses based on web of science core collection database

##### 3.2.1.1 Core journals analysis

According to Bradford’s Law, a total of 12 core journals in immune checkpoint inhibitors in endometrial cancer have been identified, such as Gynecologic Oncology, Cancers, Frontiers in Immunology, Frontiers in Oncology, and the International Journal of Gynecological Cancer (**Figure 2A**). Collectively, the 12 core journals contributed 278 articles, which account for 33.53% of the total 829 articles (**Table 2**). The following analysis will concentrate on these core journals.

**Table 2:** The core journals of the immune checkpoint inhibitor study in endometrial cancer.

Journals (WOSCC)	Number of documents (WOSCC)	Journals (PubMed)	Number of documents (PubMed)	Citations (WOSCC)	IF 2024 (WOSCC)	Q 2024 (WOSCC)	Total link strength of citation analysis (WOSCC)	Total link strength of bibliographic coupling analysis (WOSCC)
gynecologic oncology	61	gynecologic oncology	18	1218	4.1	Q1	485	44321
cancers	38	journal of clinical oncology	10	368	4.4	Q1	289	36638
frontiers in immunology	26	cancers	9	653	5.9	Q1	129	17958
frontiers in oncology	24	frontiers in immunology	7	223	3.3	Q2	174	19016
international journal of gynecological cancer	24	frontiers in oncology	6	456	4.7	Q1	230	17965
gynecologic oncology reports	20	current treatment options in oncology	6	40	1.3	Q3	79	6710
journal for immunotherapy of cancer	19	——	——	677	10.6	Q1	239	12791
international journal of molecular sciences	18	——	——	252	4.9	Q1	118	15535
journal of gynecologic oncology	14	——	——	258	3.7	Q1	100	9455
journal of clinical oncology	12	——	——	3918	41.9	Q1	794	9391
modern pathology	12	——	——	278	5.5	Q1	133	9633
clinical cancer research	10	——	——	1138	10.2	Q1	221	10554

##### 3.2.1.2 Citation and bibliographic coupling analyses of the 12 core journals

A total of 829 articles pertaining to immune checkpoint inhibitors in endometrial cancer have been published across 296 distinct journals. Among these journals, Gynecologic Oncology emerged as the leading journal with the highest publication volume (n = 61, 7.36%), followed by Cancers (n = 38, 4.58%), Frontiers in Immunology (n = 26, 3.14%), Frontiers in Oncology (n = 24, 2.90%), and the International Journal of Gynecological Cancer (n = 24, 2.90%). Collectively, the five core journals contributed 173 articles, which account for 20.87% of the total 829 articles (**Table 2**).

The citation analysis counts the number of reciprocal citations among the 12 core journals. A citation network was established for the 12 core journals (**Figure 2C**). Among these journals, Gynecologic Oncology demonstrated mutual citation relationships with the other 11 journals (links = 209). Cancers exhibited mutual citation relationships with the other 11 journals (links = 125). Frontiers in Immunology showed mutual citation relationships with the other ten journals (links = 59). Frontiers in Oncology displayed mutual citation relationships with the other 11 journals (links = 80). The International Journal of Gynecological Cancer revealed mutual citation relationships with the other 11 journals (links = 89) (**Figure 2C**).

The bibliographic coupling analysis counts the number of shared references across the 12 journals. A bibliographic coupling network was developed for the 12 journals (**Figure 2D**). Within this network, Gynecologic Oncology shared the same references with the other 11 journals (links = 13,455). Cancers shared the same references with the other 11 journals (links = 11,299). Frontiers in Immunology shared the same references with the other 11 journals (links = 5,823). Frontiers in Oncology shared the same references with the other 11 journals (links = 6,501). The International Journal of Gynecological Cancer shared the same references with the other 11 journals (links = 6,240) (**Figure 2D**).

##### 3.2.1.3 The influence analysis of journals

The number of citations, the total link strength of citation analysis, and the total link strength of bibliographic coupling analysis are important indicators for measuring the influence of journals. The three most frequently cited journals were the Journal of Clinical Oncology, Gynecologic Oncology, and Clinical Cancer Research. The citation analysis counts the number of reciprocal citations among the 296 journals. The three journals with the highest total link strength in the citation analysis were the Journal of Clinical Oncology, Gynecologic Oncology, and Cancers. The bibliographic coupling analysis counts the number of shared references across the 296 journals. The three journals with the highest total link strength in the bibliographic coupling analysis were Gynecologic Oncology, Cancers, and Frontiers in Oncology (**Table 2**).

##### 3.2.1.4 Dual-map overlay analysis of journals

The CiteSpace software was utilized to create a dual-map overlay of journals based on Journal Citation Reports, demonstrating the relationships between the disciplinary fields of the source journals for both the citing and cited literatures. The left side of the map delineates the disciplinary fields of the source journals for the citing literatures, while the right side indicates the disciplinary fields of the source journals for the cited literatures. As demonstrated in **Figure 2E**, three distinct pathways have been identified: one is depicted in orange, while the other two are represented in green. The orange pathway shows that journals within the disciplinary field of Molecular/Biology/Immunology frequently cite articles from journals in the disciplinary field of Molecular/Biology/Genetics (z-score = 2.43). Additionally, the two green pathways display that journals within the disciplinary field of Medicine/Medical/Clinical frequently cite articles from journals in the disciplinary fields of Molecular/Biology/Genetics (z-score = 5.28) and Health/Nursing/Medicine (z-score = 2.75).

#### 3.2.2 Journal analyses based on PubMed database

For the PubMed database, this research involved an analysis of 279 publications disseminated across 120 journals. Among these 279 articles, 153 are published in journals with an impact factor of 3 or higher in 2024. Among these 153 articles, according to Bradford’s Law, six core journals on immune checkpoint inhibitors in endometrial cancer have been identified: Gynecologic Oncology, Journal of Clinical Oncology, Cancers, Frontiers in Immunology, Frontiers in Oncology, and Current Treatment Options in Oncology (**Figure 2B**). Collectively, the six core journals contributed 56 articles, which account for 36.6% of the total 153 articles (**Table 2**).

To sum up, Gynecologic Oncology was the most influential journal. Cancers, Frontiers in Immunology, Frontiers in Oncology, and the Journal of Clinical Oncology were also identified as significant influential journals on immune checkpoint inhibitors in endometrial cancer.

### 3.3 Author analyses

#### 3.3.1 Author analyses based on web of science core collection database

##### 3.3.1.1 Core authors analysis

A total of 5,145 authors participated in research on immune checkpoint inhibitors in endometrial cancer. Among these authors, Makker Vicky emerged as the most prolific author, followed by Lorusso Domenica, and Pignata Sandro. The criteria for the networks among authors were specified as follows: the minimum number of documents for an author was set at eight, and the minimum number of citations for an author was set at 140. As a result, we identified ten core authors for the following constructed networks (**Table 3**).

**Table 3:** Top ten authors of the immune checkpoint inhibitor study in endometrial cancer.

Authors (WOSCC)	Number of documents (WOSCC)	Citations (WOSCC)	Total link strength of co-authorship analysis (WOSCC)	Total link strength of citation analysis (WOSCC)	Total link strength of bibliographic coupling analysis (WOSCC)
makker vicky	21	1304	277	4258	139776
lorusso domenica	19	211	184	2413	180018
pignata sandro	15	439	184	1892	142024
mills anne m.	13	444	71	1169	85170
oaknin ana	11	888	159	3645	95644
orlowski robert	9	479	117	2258	53771
ring kari l.	9	264	26	1011	71360
ross jeffrey s.	8	621	99	776	33939
mathews cara	8	490	90	2054	56828
musacchio lucia	8	145	63	1137	98702

##### 3.3.1.2 Co-authorship, citation, and bibliographic coupling analyses of the ten core authors

**Figure 4:**
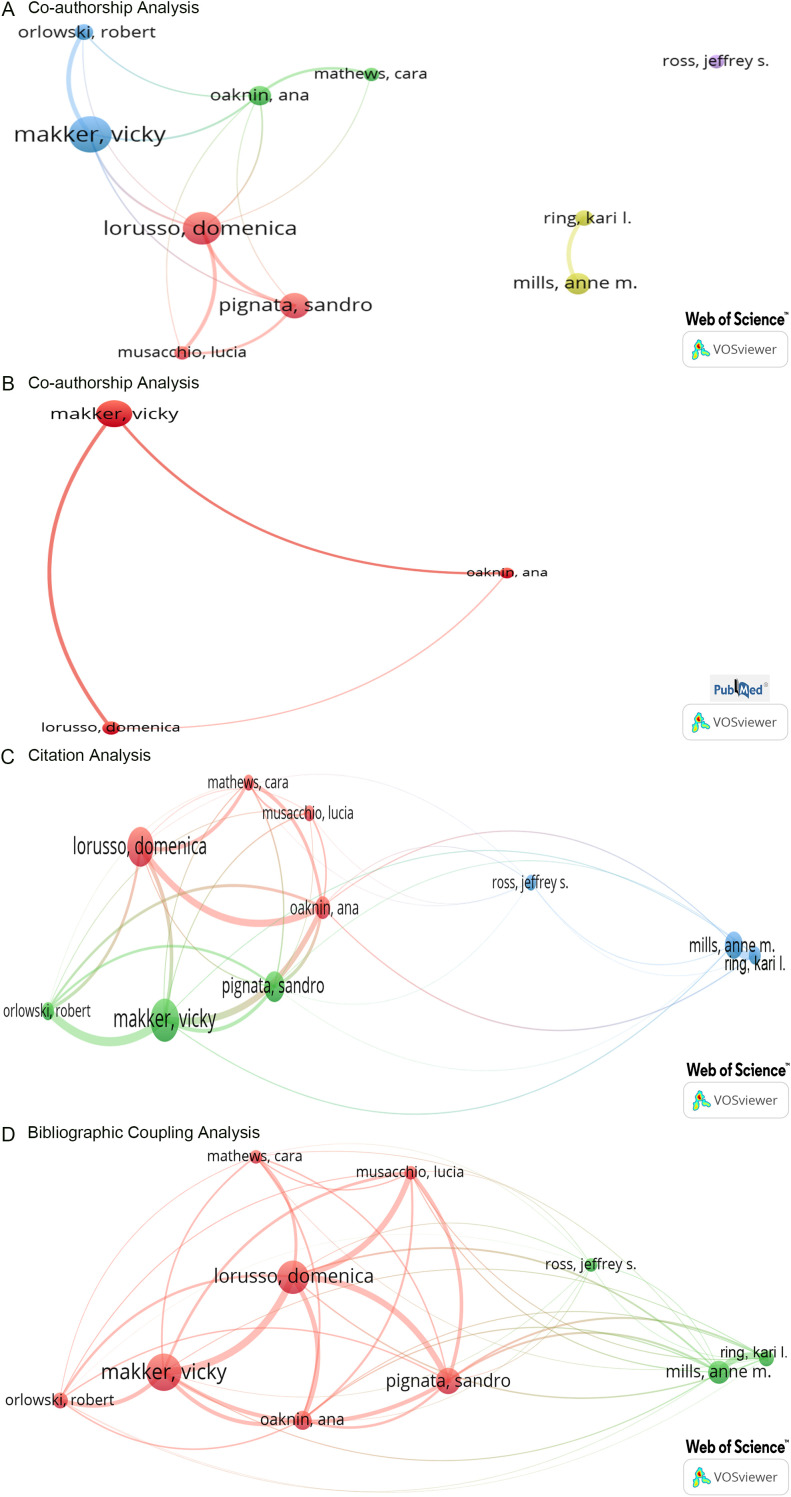
Author analyses. The dimensions of each node correspond to the quantity of documents, the thickness of the lines reflects the intensity of interactions, and the colors signify distinct clusters. **(A, B)**. The network represents the analysis of co-authorship among the core authors. **(A)** The data are obtained from the Web of Science database. **(B)** The data are obtained from the PubMed database. **(C, D)**. The data are obtained from the Web of Science database. The two networks represent the analyses of citation and bibliographic coupling for the ten core authors, respectively.

The co-authorship analysis reveals the relationships among the ten core authors by counting the number of articles they have collaboratively authored. A co-authorship network was constructed for the ten authors (**Figure 4A**). Among these authors, Makker Vicky exhibited co-authorships with the other four authors (links = 14). Lorusso Domenica showed co-authorships with the other six authors (links = 17). Pignata Sandro displayed co-authorships with the other four authors (links = 12) (**Figure 4A**).

The citation analysis counts the number of reciprocal citations among the ten authors. A citation network was established for the ten core authors (**Figure 4C**). In this network, Makker Vicky exhibited mutual citation relationships with the other eight authors (links = 112). Lorusso Domenica showed mutual citation relationships with the other seven authors (links = 85). Pignata Sandro displayed mutual citation relationships with the other nine authors (links = 60) (**Figure 4C**).

The bibliographic coupling analysis counts the number of shared references across the 5,145 authors. A bibliographic coupling network was developed for the ten core authors (**Figure 4D**). Within this network, Makker Vicky shared the same references with the other nine authors (links = 4,744). Lorusso Domenica shared the same references with the other nine authors (links = 3,680). Pignata Sandro shared the same references with the other nine authors (links = 1,664) (**Figure 4D**).

##### 3.3.1.3 The influence analysis of authors

The number of citations, the total link strength of co-authorship analysis, the total link strength of citation analysis, and the total link strength of bibliographic coupling analysis are important indicators for measuring the influence of authors. The three most frequently cited authors were Makker Vicky, Oaknin Ana, and Ross Jeffrey S. The co-authorship analysis investigates the relationships among the 5,145 authors by counting the number of articles they have co-authored. The three authors with the highest total link strength in the co-authorship analysis were Makker Vicky, Lorusso Domenica, and Pignata Sandro. The citation analysis counts the number of reciprocal citations among the 5,145 authors. The three authors with the highest total link strength in the citation analysis were Makker Vicky, Oaknin Ana, and Lorusso Domenica. The bibliographic coupling analysis counts the number of shared references among the 5,145 authors. The three authors with the highest total link strength in the bibliographic coupling analysis were Lorusso Domenica, Pignata Sandro, and Makker Vicky (**Table 3**).

#### 3.3.2 Author analyses based on PubMed database

A total of 1,611 authors participated in research on immune checkpoint inhibitors in endometrial cancer. Among these authors, Makker Vicky emerged as the most prolific author, followed by Lorusso Domenica, and Oaknin Ana. The criteria for the networks among authors were specified as follows: the minimum number of documents for an author was set at eight (**Figure 4B**).

In conclusion, Makker Vicky was the most influential author. Lorusso Domenica was also identified as relatively influential author on immune checkpoint inhibitors in endometrial cancer.

### 3.4 Institution analyses

#### 3.4.1 Institution analyses based on web of science core collection database

##### 3.4.1.1 Core institutions analysis

The research on immune checkpoint inhibitors in endometrial cancer encompassed a total of 1,535 institutions. Among these institutions, the leading four contributors to publication volume were Memorial Sloan Kettering Cancer Center, the University of Texas MD Anderson Cancer Center, Merck & Company, Incorporated, and Ohio State University. The criteria for the networks among institutions were specified as follows: the minimum number of documents for an institution was set at 13, and the minimum number of citations for an institution was set at 700. As a result, we identified ten core institutions for the following constructed networks (**Table 4**).

**Table 4:** Top ten institutions of the immune checkpoint inhibitor study in endometrial cancer.

Institutions (PubMed)	Number of documents (PubMed)	Institutions (WOSCC)	Number of documents (WOSCC)	Citations (WOSCC)	Total link strength of co-authorship analysis (WOSCC)	Total link strength of citation analysis (WOSCC)	Total link strength of bibliographic coupling analysis (WOSCC)
mem sloan kettering canc ctr	16	mem sloan kettering canc ctr	37	5520	322	4943	155289
canadian agency for drugs and technologies in health	8	univ texas md anderson canc ctr	29	3133	124	2105	99083
univ calif	7	merck & co inc	23	3558	223	3299	68709
univ texas md anderson canc ctr	7	ohio state univ	23	2796	173	3024	102582
dana farber canc inst	5	univ virginia	19	730	74	1207	57382
new york univ	5	dana farber canc inst	17	2204	137	1741	46603
johns hopkins univ sch med	4	harvard med sch	16	2161	73	1328	49675
ohio state univ	4	seoul natl univ	16	2610	134	2060	40373
washington univ	4	eisai inc	13	859	159	1689	35516
yale univ	4	massachusetts gen hosp	13	1166	71	870	24464

mem sloan kettering canc ctr, Memorial Sloan Kettering Cancer Center; univ texas md anderson canc ctr, University of Texas MD Anderson Cancer Center; merck & co inc, Merck & Company, Incorporated; ohio state univ, Ohio State University; univ virginia, University of Virginia; dana farber canc inst, Dana-Farber Cancer Institute; harvard med sch, Harvard Medical School; seoul natl univ, Seoul National University; eisai inc, Eisai Incorporated; massachusetts gen hosp, Massachusetts General Hospital; univ calif, University of California; new york univ, New York University; johns hopkins univ sch med, Johns Hopkins University School of Medicine; washington univ, Washington University; yale univ, Yale University.

##### 3.4.1.2 Co-authorship, citation, and bibliographic coupling analyses of the ten core institutions

**Figure 5:**
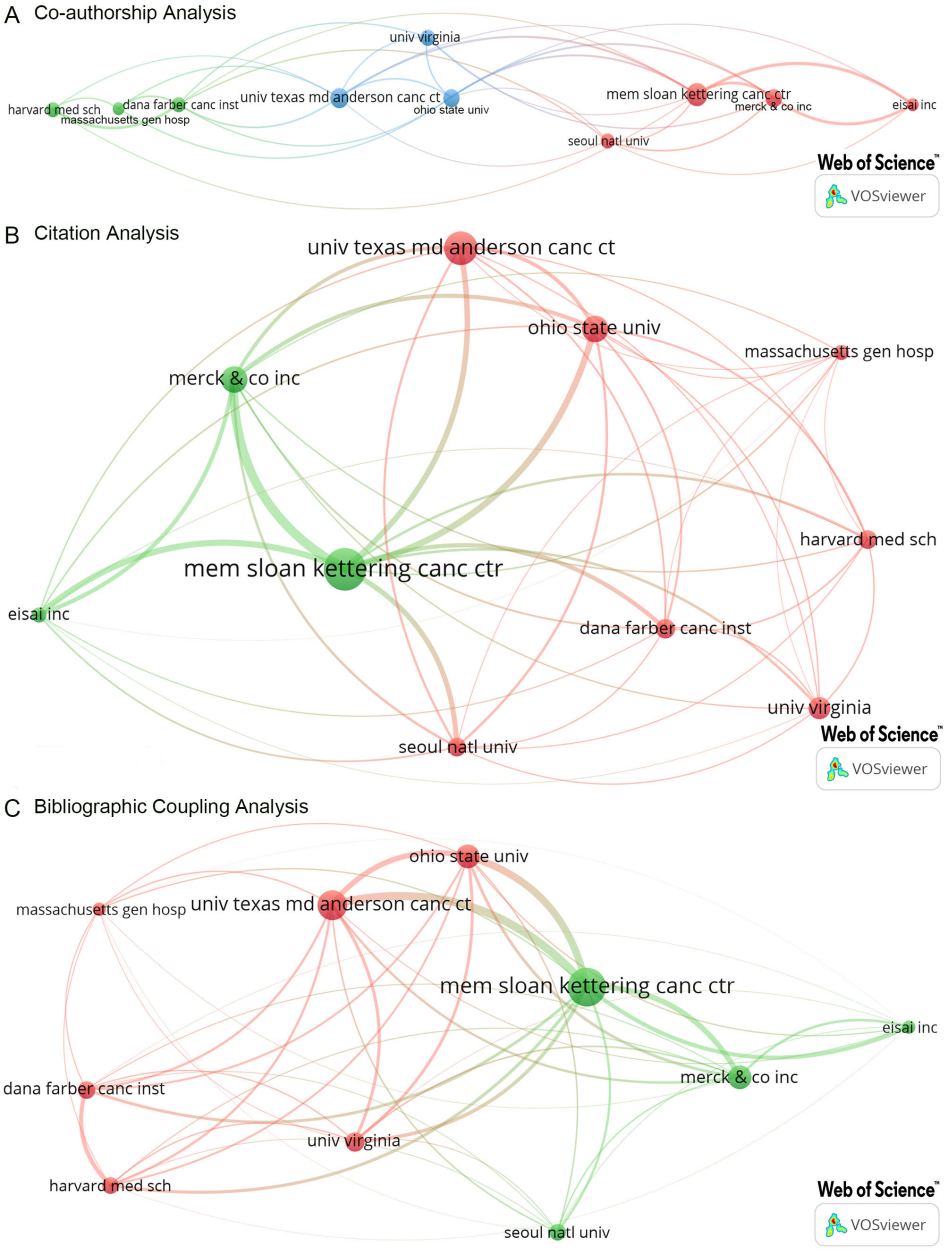
Institution analyses. The data are obtained from the Web of Science database. The three networks depict the analyses of co-authorship, citation, and bibliographic coupling pertaining to the ten core institutions, respectively. The dimensions of each node correspond to the quantity of documents, the thickness of the lines reflects the intensity of interactions, and the colors signify distinct clusters. mem sloan kettering canc ctr, Memorial Sloan Kettering Cancer Center; univ texas md anderson canc ctr, University of Texas MD Anderson Cancer Center; merck & co inc, Merck & Company, Incorporated; ohio state univ, Ohio State University; univ virginia, University of Virginia; dana farber canc inst, Dana-Farber Cancer Institute; harvard med sch, Harvard Medical School; seoul natl univ, Seoul National University; eisai inc, Eisai Incorporated; massachusetts gen hosp, Massachusetts General Hospital.

The co-authorship analysis reveals the relationships among the ten core institutions by counting the number of articles they have collaboratively authored. A co-authorship network was constructed for the ten institutions (**Figure 5A**). Among these institutions, Memorial Sloan Kettering Cancer Center demonstrated co-authorships with the other eight institutions (links = 34). The University of Texas MD Anderson Cancer Center exhibited co-authorships with the other eight institutions (links = 18). Merck & Company, Incorporated showed co-authorships with the other six institutions (links = 29). Ohio State University displayed co-authorships with the other eight institutions (links = 20) (**Figure 5A**).

The citation analysis counts the number of reciprocal citations among the ten institutions. A citation network was established for the ten institutions (**Figure 5B**). In this network, Memorial Sloan Kettering Cancer Center demonstrated mutual citation relationships with the other nine institutions (links = 416). The University of Texas MD Anderson Cancer Center exhibited mutual citation relationships with the other nine institutions (links = 208). Merck & Company, Incorporated showed mutual citation relationships with the other nine institutions (links = 281). Ohio State University displayed mutual citation relationships with the other nine institutions (links = 239) (**Figure 5B**).

The bibliographic coupling analysis counts the number of shared references across the ten institutions. A bibliographic coupling network was developed for the ten institutions (**Figure 5C**). Within this network, Memorial Sloan Kettering Cancer Center shared the same references with the other nine institutions (links = 10,281). The University of Texas MD Anderson Cancer Center shared the same references with the other nine institutions (links = 7,434). Merck & Company, Incorporated shared the same references with the other nine institutions (links = 5,009). Ohio State University shared the same references with the other nine institutions (links = 7,077) (**Figure 5C**).

##### 3.4.1.3 The influence analysis of institutions

The number of citations, the total link strength of co-authorship analysis, the total link strength of citation analysis, and the total link strength of bibliographic coupling analysis are important indicators for assessing the influence of institutions. The three most frequently cited institutions were Memorial Sloan Kettering Cancer Center, Merck & Company, Incorporated, and the University of Texas MD Anderson Cancer Center. The co-authorship analysis reveals the relationships among the 1,535 institutions by counting the number of articles they have co-authored. The three institutions with the highest total link strength in the co-authorship analysis were Memorial Sloan Kettering Cancer Center, Merck & Company, Incorporated, and Ohio State University. The citation analysis counts the number of reciprocal citations among the 1,535 institutions. The three institutions with the highest total link strength in the citation analysis were Memorial Sloan Kettering Cancer Center, Merck & Company, Incorporated, and Ohio State University. The bibliographic coupling analysis counts the number of shared references among the 1,535 institutions. The three institutions with the highest total link strength in the bibliographic coupling analysis were Memorial Sloan Kettering Cancer Center, Ohio State University, and the University of Texas MD Anderson Cancer Center (**Table 4**).

#### 3.4.2 Institution analyses based on PubMed database

For the PubMed database, this research involved an analysis of 279 publications disseminated across 974 institutions. Among these 974 institutions, 184 were first institutional affiliations. Microsoft Office Excel 2019 was used to count the number of articles published by these 184 first institutional affiliations. Among these institutions, the leading four contributors to publication volume were Memorial Sloan Kettering Cancer Center, Canadian Agency for Drugs and Technologies in Health, University of California, and the University of Texas MD Anderson Cancer Center (**Table 4**).

Overall, Memorial Sloan Kettering Cancer Center was the most influential institution. The University of Texas MD Anderson Cancer Center and Ohio State University were also identified as relatively influential institutions in immune checkpoint inhibitors for endometrial cancer.

### 3.5 Country analyses

#### 3.5.1 Country analyses based on web of science core collection database

##### 3.5.1.1 Core countries analysis

The research on immune checkpoint inhibitors in endometrial cancer encompassed a total of 63 countries. The leading three contributors to publication volume were United States, China, and Italy. The criteria for the networks among countries were specified as follows: the minimum number of documents for a country was set at 30, and the minimum number of citations for a country was set at 2,000. As a result, we identified ten core countries for the following constructed networks (**Table 5**).

**Table 5:** Top ten countries in the immune checkpoint inhibitor study of endometrial cancer.

Countries (WOSCC)	Number of documents (WOSCC)	Citations (WOSCC)	Total link strength of co-authorship analysis (WOSCC)	Total link strength of citation analysis (WOSCC)	Total link strength of bibliographic coupling analysis (WOSCC)
United States	328	15674	381	7230	311660
China	169	2093	69	2510	166515
Italy	91	6664	268	3854	161005
Japan	85	3752	165	2061	82738
England	68	3673	270	2912	96754
Canada	46	4818	236	2869	76513
France	46	6231	219	2750	77655
South Korea	44	4130	168	1972	58177
Spain	43	5336	190	2837	73422
Netherlands	30	2546	100	952	41314

##### 3.5.1.2 Co-authorship, citation, and bibliographic coupling analyses of the ten core countries

**Figure 6:**
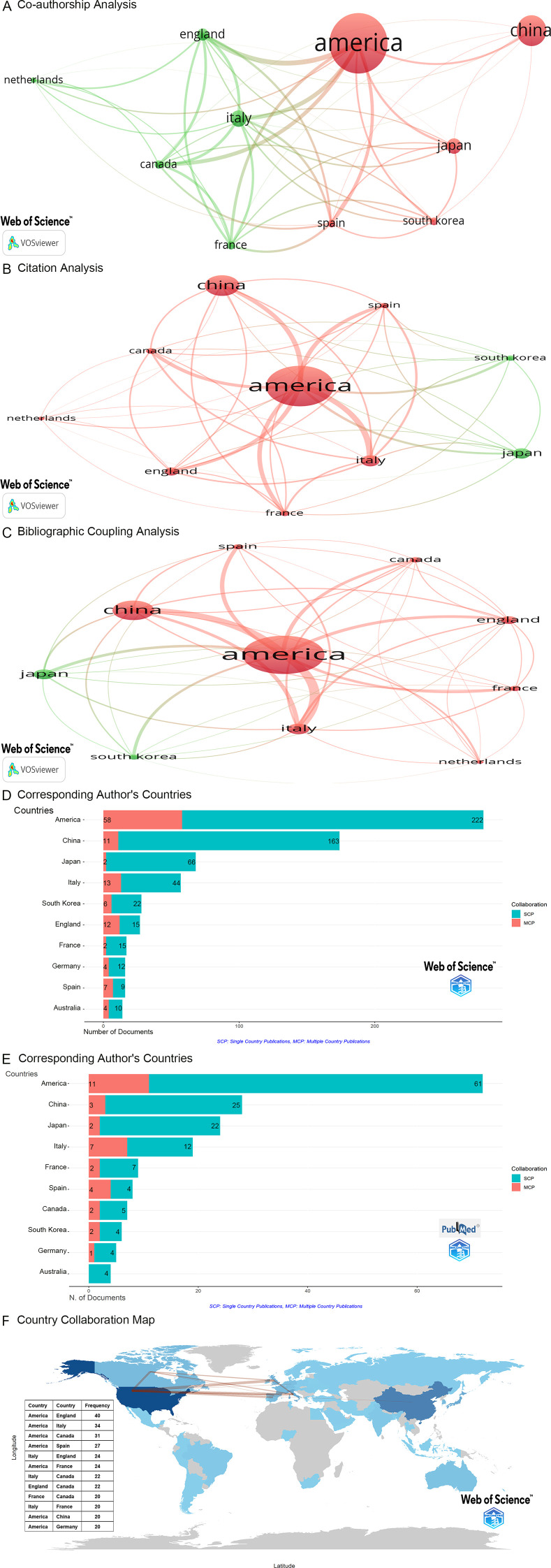
Country analyses. **(A–D)**. The data are obtained from the Web of Science database. **(A–C)**. The three networks illustrate the analyses of co-authorship, citation, and bibliographic coupling in relation to the ten core institutions, respectively. The dimensions of each node correspond to the quantity of documents, the thickness of the lines reflects the intensity of interactions, and the colors signify distinct clusters. **(D)**. The bar chart provides a statistical analysis of the countries linked to the corresponding authors of a total of 829 documents. **(E)**. The data are obtained from the PubMed database. The bar chart provides a statistical analysis of the countries linked to the corresponding authors of a total of 279 documents. **(F)** The data are obtained from the Web of Science database. A collaboration map among countries. The thickness of the red lines illustrates the degree of cooperation among countries. Various shades of blue denote the quantity of articles published by each country, with darker shades signifying a greater volume of publications. America, United States; MCP, multiple country publications; SCP, single country publications.

The co-authorship analysis reveals the relationships among the ten core countries by counting the number of articles they have collaboratively authored. A co-authorship network was constructed for the ten countries (**Figure 6A**). Among these countries, United States demonstrated co-authorships with the other nine countries (links = 214). China exhibited co-authorships with the other eight countries (links = 34). Italy showed co-authorships with the other nine countries (links = 153) (**Figure 6A**).

The citation analysis counts the number of reciprocal citations among the ten countries. A citation network was established for the ten countries (**Figure 6B**). In this network, United States demonstrated mutual citation relationships with the other nine countries (links = 4,789). China exhibited mutual citation relationships with the other nine countries (links = 1,940). Italy showed mutual citation relationships with the other nine countries (links = 2,633) (**Figure 6B**).

The bibliographic coupling analysis counts the number of shared references across the ten countries. A bibliographic coupling network was developed for the ten countries (**Figure 6C**). Within this network, United States shared the same references with the other nine countries (links = 200,845). China shared the same references with the other nine countries (links = 114,854). Italy shared the same references with the other nine countries (links = 109,583) (**Figure 6C**).

##### 3.5.1.3 The influence analysis of countries

The number of citations, the total link strength of co-authorship analysis, the total link strength of citation analysis, the total link strength of bibliographic coupling analysis, multiple country publications (MCP), single country publications (SCP), and international partnerships among countries are important indicators for evaluating the influence of countries.

The three most frequently cited countries were United States, Italy, and France. The co-authorship analysis reveals the relationships among the 63 countries by counting the number of articles they have co-authored. The three countries with the highest total link strength in the co-authorship analysis were United States, England, and Italy. The citation analysis counts the number of reciprocal citations among the 63 countries. The three countries with the highest total link strength in the citation analysis were United States, Italy, and England. The bibliographic coupling analysis counts the number of shared references among the 63 countries. The three countries with the highest total link strength in the bibliographic coupling analysis were United States, China, and Italy (**Table 5**).

Based on the analyses of multiple country publications (MCP), United States (n = 58) was recognized as the primary contributor, followed by Italy (n = 13) and England (n = 12). In contrast, the analyses of single country publications (SCP) indicated that United States (n = 222) was the foremost contributor, followed by China (n = 163) and Japan (n = 66) (**Figure 6D**).

A country’s collaboration world map was visualized, with the minimum edges set at 20. United States was the most influential country, exhibiting international partnerships with seven other countries worldwide (Frequency = 196). Notably, United States maintained the most significant degree of collaboration with England, followed by Italy and Canada. Italy exhibited international partnerships with four other countries worldwide (Frequency = 100). Canada exhibited international partnerships with four other countries worldwide (Frequency = 95) (**Figure 6F**).

#### 3.5.2 Country analyses based on PubMed database

For the PubMed database, this research involved an analysis of 279 publications from 46 different countries. Based on the analyses of multiple country publications (MCP), United States (n = 11) was recognized as the primary contributor, followed by Italy (n = 7) and Spain (n = 4). In contrast, the analyses of single country publications (SCP) indicated that United States (n = 61) was the foremost contributor, followed by China (n = 25) and Japan (n = 22) (**Figure 6E**).

All in all, United States was the most influential country. Italy, China, England, and Japan were also identified as relatively influential countries in the study of immune checkpoint inhibitors for endometrial cancer.

### 3.6 Author keyword analyses

#### 3.6.1 Author keyword analyses based on web of science core collection database

##### 3.6.1.1 Annual thematic evolution analysis of author keywords

**Figure 7:**
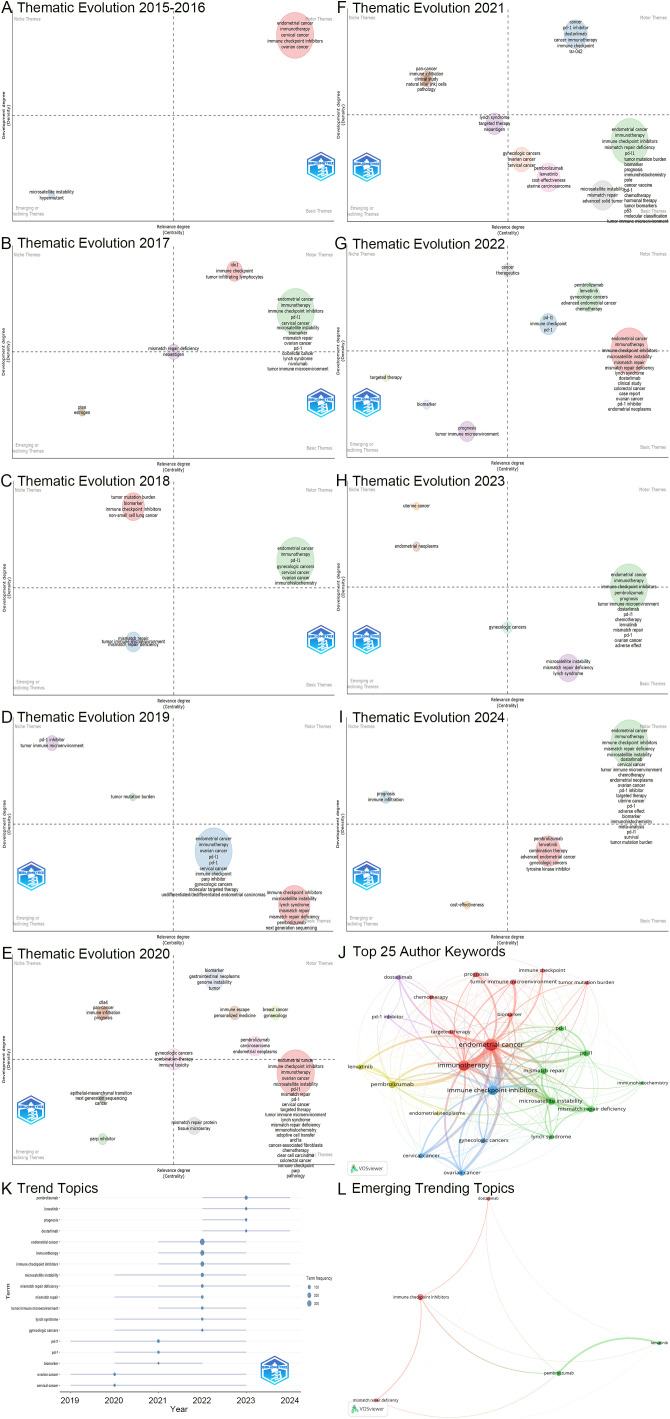
Author keyword analyses. The data are obtained from the Web of Science database. **(A-I)** The annual thematic evolution maps of author keywords demonstrate the development and interrelationships of themes from 2015 to 2024. **(J)** The co-occurrence network was developed for the top 25 most frequent thematic keywords. **(K)** The trend topic map is based on author keywords from 2015 to 2024. **(L)** The co-occurrence network map of five emerging trending topics. pd-l1: programmed cell death ligand 1 (PD-L1); pd-1: programmed death receptor 1 (PD-1).

Bibliometrix was utilized to conduct annual thematic evolution analyses of author keywords. Since only one article was published in 2015, we analyzed the articles published in 2015 and 2016 together. **Figures 7A–I** depict the annual thematic evolution analyses of author keywords, which demonstrate the development and interrelationships of themes from 2015 to 2024, resulting in a total of 82 thematic keywords (**Supplementary File 3**). **Table 6** represents the annual thematic evolution of the top 25 most frequent thematic keywords. The keywords that have remained Motor Themes from 2022 to 2024 and have persisted as themes from 2015 to 2024 include endometrial cancer, immunotherapy, and immune checkpoint inhibitors. The immune checkpoint inhibitors study in endometrial cancer remains a research hotspot in recent years (2022-2024). Except for 2018, microsatellite instability has emerged as a theme each year from 2015 to 2024, serving as the Motor Theme in 2024. Mismatch repair deficiency has emerged as a theme each year from 2017 to 2024, serving as the Motor Theme in 2024. Except for 2018, programmed death receptor 1 (PD-1) has emerged as a theme each year from 2017 to 2024, serving as the Motor Theme from 2022 to 2024. Programmed cell death ligand 1 (PD-L1) has emerged as a theme each year from 2017 to 2024, serving as the Motor Theme from 2022 to 2024. Chemotherapy has emerged as a theme each year from 2020 to 2024, serving as the Motor Theme from 2022 to 2024. Pembrolizumab has emerged as a theme each year from 2019 to 2024. Lenvatinib has emerged as a theme each year from 2021 to 2024. Dostarlimab has emerged as a Motor Theme each year from 2021 to 2024. Microsatellite instability, mismatch repair deficiency, PD-1, PD-L1, chemotherapy, pembrolizumab, lenvatinib, and dostarlimab remain research hotspots in the study of immune checkpoint inhibitors in endometrial cancer in recent years.

**Table 6:** Top 25 most frequent thematic keywords of the immune checkpoint inhibitor study in endometrial cancer.

Keyword	Frequency (WOSCC)	Occurrences (WOSCC)	Occurrences (PubMed)	Total link strength (WOSCC)	Thematic evolution (WOSCC)								
					2015-2016	2017	2018	2019	2020	2021	2022	2023	2024
endometrial cancer	9	350	119	1569	Motor	Motor	Motor	Basic	Basic	Basic	Motor	Motor	Motor
immunotherapy	9	224	71	1024	Motor	Motor	Motor	Basic	Basic	Basic	Motor	Motor	Motor
immune checkpoint inhibitors	9	184	74	859	Motor	Motor	Niche	Basic	Basic	Basic	Motor	Motor	Motor
ovarian cancer	9	52	19	253	Motor	Motor	Motor	Basic	Basic	Basic	Motor	Motor	Motor
microsatellite instability	8	97	27	480	Emerging	Motor	——	Basic	Basic	Basic	Motor	Basic	Motor
mismatch repair deficiency	8	64	15	315	——	Emerging	Emerging	Basic	Basic	Basic	Motor	Basic	Motor
PD-L1	8	64	14	305	——	Motor	Motor	Basic	Basic	Basic	Motor	Motor	Motor
tumor immune microenvironment	8	47	9	236	——	Motor	Declining	Niche	Basic	Basic	Declining	Motor	Motor
mismatch repair	7	55	15	262	——	Motor	Declining	Basic	Basic	Basic	Motor	Motor	——
PD-1	7	43	17	231	——	Motor	——	Basic	Basic	Basic	Motor	Motor	Motor
cervical cancer	7	40	19	218	Motor	Motor	Motor	Basic	Basic	Basic	——	——	Motor
gynecologic cancers	7	38	12	168	——	——	Motor	Basic	Intersection	Basic	Motor	Basic	Basic
pembrolizumab	6	97	50	439	——	——	——	Basic	Motor	Basic	Motor	Motor	Basic
lynch syndrome	6	40	15	190	——	Motor	——	Basic	Basic	Declining	Motor	Basic	——
biomarker	6	33	5	150	——	Motor	Niche	——	Motor	Basic	Declining	——	Motor
prognosis	5	45	2	195	——	——	——	——	Niche	Basic	Declining	Motor	Niche
chemotherapy	5	26	14	139	——	——	——	——	Basic	Basic	Motor	Motor	Motor
immune checkpoint	5	24	3	114	——	Motor	——	Basic	Basic	Motor	Motor	——	——
lenvatinib	4	47	24	228	——	——	——	——	——	Basic	Motor	Motor	Basic
dostarlimab	4	36	13	167	——	——	——	——	——	Motor	Motor	Motor	Motor
targeted therapy	4	29	11	166	——	——	——	——	Basic	Declining	Declining	——	Motor
PD-1 inhibitor	4	28	17	126	——	——	——	Niche	——	Motor	Motor	——	Motor
tumor mutation burden	4	24	3	110	——	——	Niche	Niche	——	Basic	——	——	Motor
endometrial neoplasms	4	22	9	65	——	——	——	——	Motor	——	Motor	Niche	Motor
immunohistochemistry	4	18	—	75	——	——	Motor	——	Basic	Basic	——	——	Motor

PD-L1, programmed cell death ligand 1; PD-1, programmed death receptor 1.

##### 3.6.1.2 Co-occurrence analysis of author keywords

VOSviewer was employed to identify 1,123 author keywords. For each keyword, the frequency of occurrence was assessed, and the total strength of co-occurrence links with other keywords was also calculated. A **co-occurrence network** was developed for the top 25 most frequent thematic keywords (**Figure 7J**). **Table 6** lists the frequency of occurrences and the total strength of co-occurrence links of the 25 keywords. Among these keywords, endometrial cancer exhibited co-occurrence relationships with the other 23 keywords (links = 759). Immunotherapy exhibited co-occurrence relationships with the other 24 keywords (links = 524). Immune checkpoint inhibitors exhibited co-occurrence relationships with the other 24 keywords (links = 437). Microsatellite instability exhibited co-occurrence relationships with the other 23 keywords (links = 258). Mismatch repair deficiency exhibited co-occurrence relationships with the other 20 keywords (links = 167). PD-1 exhibited co-occurrence relationships with the other 20 keywords (links = 131). PD-L1 exhibited co-occurrence relationships with the other 22 keywords (links = 173). Chemotherapy exhibited co-occurrence relationships with the other 19 keywords (links = 72). Pembrolizumab exhibited co-occurrence relationships with the other 21 keywords (links = 211). Lenvatinib exhibited co-occurrence relationships with the other 15 keywords (links = 108). Dostarlimab exhibited co-occurrence relationships with the other 12 keywords (links = 87) (**Figure 7J**).

##### 3.6.1.3 Trend topic analysis of author keywords

Bibliometrix was employed to perform a trend topic analysis based on author keywords, utilizing time series data from 2015 to 2024. The objective was to identify emerging trending topics in the study of immune checkpoint inhibitors for endometrial cancer. **Figure 7K** shows each trend topic as a line, where the length represents the duration, and the size of the circle reflects the frequency of the keywords’ occurrence. Pembrolizumab, lenvatinib, dostarlimab, immune checkpoint inhibitors, and mismatch repair deficiency have been consistently popular trend topics from 2022 to 2024 (**Figure 7K**).

VOSviewer was utilized to construct a co-occurrence network for the five emerging trending topics (**Figure 7L**). Among these topics, pembrolizumab exhibited co-occurrence relationships with the other four keywords (links = 79). Lenvatinib exhibited co-occurrence relationships with the other four keywords (links = 54). Dostarlimab exhibited co-occurrence relationships with the other four keywords (links = 23). Immune checkpoint inhibitors exhibited co-occurrence relationships with the other four keywords (links = 58). Mismatch repair deficiency exhibited co-occurrence relationships with the other four keywords (links = 30) (**Figure 7L**).

#### 3.6.2 Author keyword analyses based on PubMed database

For the PubMed database, VOSviewer was employed to identify 423 author keywords. For each keyword, the frequency of occurrence was assessed. Among the 25 author keywords from the WoSCC database in the previous text, except for immunohistochemistry, the other 24 author keywords were also important in the data analysis of the PubMed database (**Table 6**).

### 3.7 Reference analyses based on web of science core collection database

#### 3.7.1 Co-citation analysis of references

**Figure 8:**
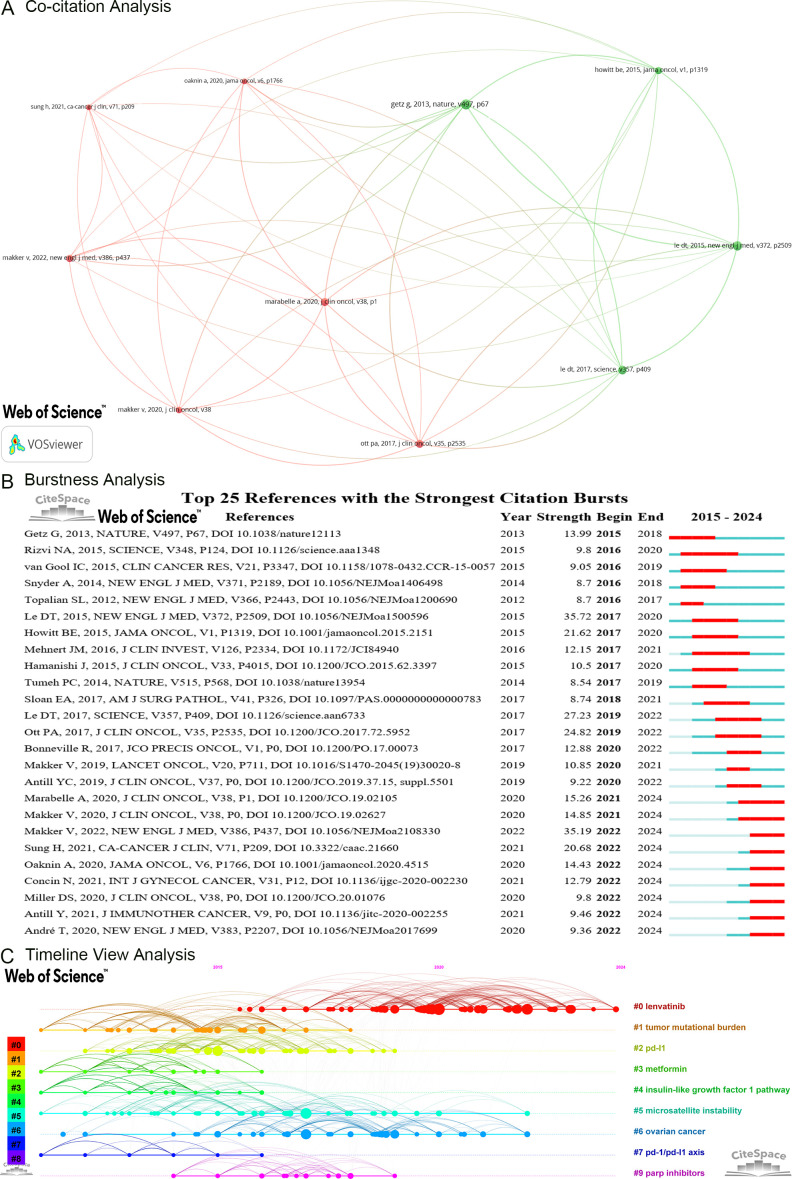
Reference analyses. The data are obtained from the Web of Science database. **(A)** The network depicts the co-citation analysis of the ten most frequently co-cited references. The dimensions of each node correspond to the citation frequency of the references, while the thickness of the lines represents the intensity of interactions. Additionally, the colors denote distinct clusters. **(B)** The top 25 references demonstrating the strongest citation bursts. **(C)** The analysis of references presented in the timeline view, as generated by CiteSpace, employs the log-likelihood ratio method. new engl j med, New England Journal of Medicine; j clin oncol, Journal of Clinical Oncology; jama oncol, JAMA Oncology; ca-cancer j clin, CA-A Cancer Journal for Clinicians; IF, Impact Factor; Q, Journal Citation Reports Quartile; PD-L1, programmed cell death ligand 1; PD-1, programmed death receptor 1.

In the last ten years, a total of 24,043 references related to immune checkpoint inhibitors in endometrial cancer have been co-cited across 829 articles. The three references with the highest total link strength in the co-citation analysis were “Getz G, 2013,” “Le Dt, 2015,” and “Le Dt, 2017.” The three most frequently cited references were “Getz G, 2013,” “Le Dt, 2015,” and “Le Dt, 2017.” Each of the top ten references was cited at least 100 times, with three references exceeding 200 citations (**Table 7**). A co-citation network was visualized for the ten most cited references (**Figure 8A**). Among these references, “Getz G, 2013” showed the strongest co-citation relationships with the other nine references (links = 710). “Le Dt, 2015” showed the co-citation relationships with the other nine references (links = 542). “Le Dt, 2017” showed the co-citation relationships with the other nine references (links = 524) (**Figure 8A**).

**Table 7:** Top ten most cited references of the immune checkpoint inhibitor study in endometrial cancer. .

Top ten most cited references (WOSCC)	Citations (WOSCC)	Total link strength of co-citation analysis (WOSCC)	First author (WOSCC)	Year (WOSCC)	Journal (WOSCC)	IF(2024) (WOSCC)	Q (WOSCC)
Integrated genomic characterization of endometrial carcinoma	276	17354	getz g	2013	nature	48.5	Q1
PD-1 Blockade in Tumors with Mismatch-Repair Deficiency	242	14875	le dt	2015	new engl j med	78.5	Q1
Mismatch repair deficiency predicts response of solid tumors to PD-1 blockade	209	13087	le dt	2017	science	45.8	Q1
Safety and Antitumor Activity of Pembrolizumab in Advanced Programmed Death Ligand 1-Positive Endometrial Cancer: Results From the KEYNOTE-028 Study	181	10863	ott pa	2017	j clin oncol	41.9	Q1
Efficacy of Pembrolizumab in Patients With Noncolorectal High Microsatellite Instability/Mismatch Repair-Deficient Cancer: Results From the Phase II KEYNOTE-158 Study	179	10651	marabelle a	2020	j clin oncol	41.9	Q1
Lenvatinib plus Pembrolizumab for Advanced Endometrial Cancer	154	7413	makker v	2022	new engl j med	78.5	Q1
Association of Polymerase e-Mutated and Microsatellite-Instable Endometrial Cancers With Neoantigen Load, Number of Tumor-Infiltrating Lymphocytes, and Expression of PD-1 and PD-L1	145	9556	howitt be	2015	jama oncol	20.1	Q1
Lenvatinib Plus Pembrolizumab in Patients With Advanced Endometrial Cancer	126	7584	makker v	2020	j clin oncol	41.9	Q1
Clinical Activity and Safety of the Anti-Programmed Death 1 Monoclonal Antibody Dostarlimab for Patients With Recurrent or Advanced Mismatch Repair-Deficient Endometrial Cancer: A Nonrandomized Phase 1 Clinical Trial	103	6255	oaknin a	2020	jama oncol	20.1	Q1
Global Cancer Statistics 2020: GLOBOCAN Estimates of Incidence and Mortality Worldwide for 36 Cancers in 185 Countries	100	7123	sung h	2021	ca-cancer j clin	232.4	Q1

new engl j med, New England Journal of Medicine; j clin oncol, Journal of Clinical Oncology; jama oncol, JAMA Oncology; ca-cancer j clin, CA-A Cancer Journal for Clinicians; IF, Impact Factor; Q, Journal Citation Reports Quartile; PD-L1, programmed cell death ligand 1; PD-1, programmed death receptor 1.

#### 3.7.2 Citation burstness analysis of references

A citation burst analysis was conducted on a dataset comprising 24,043 references, resulting in the identification of the 25 references with the most significant burst strength. **Figure 8B** illustrates the citation bursts associated with these references, where each bar corresponds to a specific year; time intervals are denoted in blue, while the periods of citation bursts are highlighted in red. Notably, “Le Dt, 2015” exhibits the strongest strength (strength = 35.72) in citation burst analysis from 2022 to 2024 (**Figure 8B**).

#### 3.7.3 Timeline view analysis of references

The cluster and timeline analyses were performed on the cited references, facilitating the identification of the most frequently occurring terms within each cluster, which were then utilized as labels for the respective clusters. As depicted in **Figure 8C**, each line represents a distinct cluster, culminating in a total of nine clusters generated through the Log-Likelihood Ratio (LLR) method. The calculated modularity Q value was found to be 0.72, while the mean silhouette value was assessed at 0.89. Notably, the cluster associated with lenvatinib (#0) was established as early as 2015 and continued to be relevant through 2024 (**Figure 8C**).

## 4 Discussion

This study aims to provide an in-depth understanding of the global status, research hotspots, recent trend topics, knowledge mapping, and future directions in the investigation of immune checkpoint inhibitors in endometrial cancer, utilizing bibliometric analysis.

### 4.1 General information and global status

For the WoSCC database, this research involved an analysis of 829 publications disseminated across 296 journals, authored by 5,145 researchers affiliated with 1,535 institutions from 63 different countries. Among the 829 articles analyzed, Marabelle (2020), Mosele (2020), Dudley (2016), Luchini (2019), Ott (2017), and Makker (2022) are the six most important articles concerning immune checkpoint inhibitors in endometrial cancer, as identified through publication, citation, and bibliographic coupling analyses. For the PubMed database, this research involved an analysis of 279 publications disseminated across 120 journals, authored by 1,611 researchers affiliated with 974 institutions from 46 different countries. The articles in question were published within the timeframe from January 1, 2015, to December 31, 2024, and are cataloged in the WoSCC or PubMed databases. The period from 2018 to 2024 witnessed the rapid development of research on immune checkpoint inhibitors in endometrial cancer. This consistent rapid growth in publications indicates a burgeoning interest in this field.

The five most influential journals in this field, determined by publication, core journals, citation, bibliographic coupling, and dual-map overlay analyses, are Gynecologic Oncology, Cancers, Frontiers in Immunology, Frontiers in Oncology, and the Journal of Clinical Oncology. All of these journals are classified above JCR Q2 and have impact factors exceeding 3. Analyses of the volume of publications and citations attributed to individual authors, alongside evaluations of core authors, co-authorship, citation, and bibliographic coupling, reveal that the two most influential authors regarding immune checkpoint inhibitors in endometrial cancer are Makker Vicky and Lorusso Domenica. Furthermore, analyses of the volume of publications and citations attributed to various institutions, along with evaluations of core institutions, co-authorship, citation, and bibliographic coupling, reveal that the three most influential institutions identified are Memorial Sloan Kettering Cancer Center, the University of Texas MD Anderson Cancer Center, and Ohio State University. Additionally, analyses of the volume of publications and citations attributed to different countries, as well as evaluations of core countries, co-authorship, citation, bibliographic coupling, multiple-country publications, single-country publications, and the countries’ collaboration world map, reveal that the five leading countries are United States, Italy, China, England, and Japan. In terms of international collaboration concerning immune checkpoint inhibitors in endometrial cancer, United States demonstrates the most significant influence and has established the most extensive network of collaborative partnerships, with United States and England forming the closest research partnership.

### 4.2 Research hotspots and emerging trending topics

The investigation of immune checkpoint inhibitors in endometrial cancer has continued to be a significant area of research and a Motor Theme over the past three years. Through in-depth analyses of keyword co-occurrence, frequency, and annual thematic evolution, we have identified that microsatellite instability, mismatch repair deficiency, PD-1, PD-L1, chemotherapy, pembrolizumab, lenvatinib, and dostarlimab remain research hotspots in this field. Through in-depth analyses of keyword co-occurrence, frequency, and trending topics, we have identified that pembrolizumab, lenvatinib, dostarlimab, immune checkpoint inhibitors, and mismatch repair deficiency have been consistently popular emerging trending topics from 2022 to 2024 in this field.

#### 4.2.1 Microsatellite instability and mismatch repair deficiency

Microsatellites are short tandem repetitive DNA sequences that are widely present in the human genome (24). Microsatellite instability (MSI) is an important research hotspot in the study of immune checkpoint inhibitors for endometrial cancer. MSI is defined as the inherent variation in the number of nucleotides within these repetitive DNA sequences, characterized by either the deletion or addition of nucleotide units (25). Such alterations can result in the shortening or lengthening of microsatellites. The core mechanism of MSI is often attributed to mismatch repair deficiency (dMMR) (24) and strand slippage misalignment during DNA replication (26). The dMMR is an important research hotspot in the study of immune checkpoint inhibitors for endometrial cancer. Mismatch repair represents a crucial mechanism essential for maintaining genomic integrity (27). When the mismatch repair (MMR) function is operating normally, strand slippage misalignments are effectively repaired. However, when MMR is deficient, the accumulation of strand slippage mutations results in MSI, which subsequently contributes to the development of cancer (28, 29). The metastasis of endometrial cancer to lymph nodes plays a critical role in shaping treatment decisions and influencing patient outcomes (2, 30). The MSI group exhibited a significantly higher incidence of occult lymph node metastasis when contrasted with the microsatellite stability (MSS) group (19% vs. 6.7%, p = 0.005). MSI was present in both early-stage and advanced-stage endometrial cancer (31). The multivariate analysis confirmed that MSI serves as an independent risk factor for the presence of occult lymph node metastasis (odds ratio = 1.105, p = 0.02) in early-stage endometrial cancer (2). MSI is likely an important biomarker associated with unfavorable prognosis in patients diagnosed with endometrioid endometrial cancer. The pooled hazard ratios (HR) for patients diagnosed with endometrioid endometrial cancer indicated that MSI was significantly correlated with shorter overall survival [HR = 1.37, p = 0.048], shorter disease-free survival [HR = 1.99, p = 0.000], and a higher recurrence rate [odds ratios = 2.72, p = 0.000] (32). Next-generation sequencing-based MSI and dMMR serve as predictive biomarkers for the efficacy of immune checkpoint inhibitors in the first-line treatment of advanced-stage endometrial cancer (33). High-frequency MSI (MSI-H)/dMMR is receiving growing attention as a potential biomarker for assessing the suitability of advanced cancer patients for treatment with immune checkpoint inhibitors (29).

#### 4.2.2 PD-1 and PD-L1

PD-1 and its ligand, PD-L1, are critical immune checkpoints (34) and significant targets for cancer immunotherapy (35). PD-1 is mainly located on the surface of immune cells (36), while PD-L1 is predominantly found on the surface of cancer cells and antigen-presenting cells (37). The combination of PD-1 and PD-L1 leads to immunosuppression by inhibiting the activation and proliferation of T cells (34). Pembrolizumab, the first immune checkpoint inhibitor targeting PD-1, received approval from the United States Food and Drug Administration (FDA) in 2014 (38). Atezolizumab, the first immune checkpoint inhibitor targeting PD-L1, received approval from the FDA in 2016 (39). Immune checkpoint inhibitors that target PD-1 or PD-L1 have received ongoing marketing approval and expanded indications from 2014 to 2024 (40). The FDA provided accelerated approval for the therapy of pembrolizumab for advanced endometrial cancer that is neither MSI-H nor dMMR in 2019 (41). The drugs approved by the FDA significantly influence the research direction of scientific researchers. Through thematic evolution analyses, we found that pembrolizumab has emerged as a significant research theme in the study of immune checkpoint inhibitors for endometrial cancer since 2019. PD-1 and PD-L1 have emerged as important research hotspots in the study of immune checkpoint inhibitors for endometrial cancer. The in-depth investigation into the PD-1/PD-L1 pathway has clarified the molecular mechanisms underlying immune checkpoint inhibitors for endometrial cancer, providing insights into potential strategies for cancer treatment.

#### 4.2.3 Pembrolizumab and lenvatinib

The FDA provided accelerated approval for the combination therapy of pembrolizumab and lenvatinib for the treatment of patients diagnosed with advanced endometrial cancer that is neither MSI-H nor dMMR in 2019 (41). This approval is specifically intended for patients who have experienced disease progression after previous systemic therapy and who are not eligible for curative surgical procedures or radiation therapy (41). Lenvatinib combined with pembrolizumab resulted in significantly longer progression-free survival (PFS) (median PFS: 6.6 vs. 3.8 months, p < 0.001) and overall survival (OS) (median OS: 17.4 vs. 12.0 months, p < 0.001) compared to chemotherapy in patients diagnosed with advanced endometrial cancer characterized by pMMR (42). Through thematic evolution analyses, we found that pembrolizumab has emerged as a significant research theme in the study of immune checkpoint inhibitors for endometrial cancer since 2019. The FDA granted regular approval to pembrolizumab in combination with lenvatinib for advanced endometrial carcinoma that is not MSI-H or dMMR in 2021 (https://www.fda.gov/). Lenvatinib, a multitargeted receptor kinase inhibitor, received its first global approval from the FDA in 2015 (43). Through thematic evolution analyses, we found that lenvatinib has emerged as a significant research theme in the study of immune checkpoint inhibitors for endometrial cancer since 2021. Pembrolizumab and lenvatinib have emerged as important research hotspots and recent trending topics in the study of immune checkpoint inhibitors for endometrial cancer through thematic evolution analyses. The drugs approved by the FDA significantly influence the research direction of scientific researchers. The thorough investigation of the mechanisms of action of immune checkpoint inhibitors in endometrial cancer, coupled with the validation of their substantial clinical benefits, has expedited the FDA approval process.

#### 4.2.4 Dostarlimab

The thematic evolution analysis shows that from 2019 to 2021, both immune checkpoint inhibitors and endometrial cancer were the Basic themes (**Table 6**). Dostarlimab, a humanized monoclonal antibody that acts as a PD-1 receptor antagonist, received its first approval from the FDA in 2021 for the treatment of adult patients with recurrent or advanced endometrial cancer characterized by dMMR (44). The approval was based on evidence from the GARNET trial (NCT02715284) (44). From 2022 to 2024, endometrial cancer, immune checkpoint inhibitors, and dostarlimab were the Motor Themes (**Table 6**). This clearly shows that drugs approved by the FDA serve as indicators guiding researchers in selecting their research directions. Dostarlimab has exhibited sustained antitumor efficacy and safety in patients with advanced or recurrent endometrial cancer characterized by dMMR/MSI-H (45). The median overall survival for dostarlimab in the GARNET cohort was longer, ranging from 27.1 to 40.5 months, compared to the real-world cohort, which had a median survival of 10.3 months (17). Through thematic evolution analyses, we found that dostarlimab has emerged as a Motor Theme and an important research hotspot in the study of immune checkpoint inhibitors for endometrial cancer since 2021. Dostarlimab and dMMR have emerged as significant recent trend topics in the study of immune checkpoint inhibitors for endometrial cancer.

#### 4.2.5 Chemotherapy

Dostarlimab received its first approval from the FDA in 2021 for recurrent or advanced endometrial cancer. The approval is based on evidence from the GARNET trial (NCT02715284), which involved 71 patients diagnosed with advanced or recurrent endometrial cancer characterized by deficient mismatch repair (dMMR). In these cases, certain types of chemotherapy were ineffective or had ceased to be effective (44). Through thematic evolution analyses, we found that chemotherapy has emerged as a Motor Theme and an important research hotspot in the study of immune checkpoint inhibitors for endometrial cancer from 2022 to 2024.

### 4.3 Future directions

With the ongoing approval and marketing of various immune checkpoint inhibitors for endometrial cancer by the FDA, the field has experienced significant growth in publications over the past decade, along with a burgeoning interest from numerous scholars. In light of the current research hotspots, emerging trending topics, and clinical needs, future research on immune checkpoint inhibitors for endometrial cancer should focus on clarifying the anti-tumor mechanisms and drug resistance principles of these inhibitors, developing new combination treatment regimens, optimizing biomarker systems, establishing dynamic efficacy prediction models, developing immune microenvironment remodeling strategies, seeking new therapeutic targets, and establishing an early warning system and hierarchical treatment plan for immune-related adverse reactions.

### 4.4 Limitations

This study is constrained by some limitations. The citation count analysis presented in this study has not been adjusted for temporal normalization. It is essential to recognize that earlier publications typically garner a greater number of citations as time progresses, potentially introducing a temporal bias that may influence the outcomes of citation and co-citation analyses. Furthermore, self-citations were neither excluded nor adjusted for in the citation and bibliographic coupling analyses. Additionally, the literature included in this analysis was confined to publications in the English language, potentially introducing a language-related bias. Finally, considering that the WoSCC and PubMed databases undergo ongoing updates, it is plausible that our study may have unintentionally missed certain recent research findings.

## 5 Conclusion

This study presents a comprehensive analysis of the scientometric characteristics related to worldwide publications concerning immune checkpoint inhibitors in the context of endometrial cancer. It outlines the knowledge frameworks, key contributors, research hotspots, emerging trending topics, and trends in innovation within this field.

1. The past decade has witnessed rapid development in the application of immune checkpoint inhibitors for the treatment of endometrial cancer. Investigations into these inhibitors within the framework of endometrial cancer have attracted considerable international attention, with United States, China, and Italy recognized as the foremost contributors to this area of research. Importantly, the highest level of international collaboration has been recorded between United States and England.
2. The journals Gynecologic Oncology, Cancers, Frontiers in Immunology, Frontiers in Oncology, and the Journal of Clinical Oncology are acknowledged as the most prominent publications in the academic literature addressing the use of immune checkpoint inhibitors in the treatment of endometrial cancer.
3. Current research hotspots in immune checkpoint inhibitors in the treatment of endometrial cancer include microsatellite instability, mismatch repair deficiency, PD-1, PD-L1, chemotherapy, pembrolizumab, lenvatinib, and dostarlimab.
4. Emerging trending topics in this field encompass pembrolizumab, lenvatinib, dostarlimab, immune checkpoint inhibitors, and mismatch repair deficiency.
5. Future research on immune checkpoint inhibitors for endometrial cancer should focus on developing new combination treatment regimens, seeking new therapeutic targets, optimizing hierarchical treatment plans, establishing dynamic efficacy prediction models, optimizing biomarker systems, establishing an early warning system for immune-related adverse reactions, and advancing precision medicine.

## Data Availability

The original contributions presented in the study are included in the article/**Supplementary Material**. Further inquiries can be directed to the corresponding author.
