# Supplementary material for: A systematic review of immune checkpoint inhibitors in endometrial cancer
Source: Front Oncol. 2026 Jun 8;16:1776831. doi: 10.3389/fonc.2026.1776831 (PMC13284135; doi:10.3389/fonc.2026.1776831)
Supplement: Supplementary file 1 [file Table1.docx]

**Supplementary File 1.Search Strategy of Web of Science Database**

| Search strategy | Term | Date | Retrieval results |
| --- | --- | --- | --- |
| Query #1 | ((((((((((((((((((((TS=(Endometrial Neoplasm)) or TS=(Neoplasm, Endometrial)) or TS=(Neoplasms, Endometrial)) or TS=(Endometrial Carcinoma)) or TS=(Carcinoma, Endometrial)) or TS=(Carcinomas, Endometrial)) or TS=(Endometrial Carcinomas)) or TS=(Cancer of Endometrium)) or TS=(Endometrium Cancers)) or TS=(Endometrium Cancer)) or TS=(Cancer, Endometrium)) or TS=(Cancers, Endometrium)) or TS=(Cancer of the Endometrium)) or TS=(Carcinoma of Endometrium)) or TS=(Endometrium Carcinoma)) or TS=(Endometrium Carcinomas)) or TS=(Endometrial Cancer)) or TS=(Cancer, Endometrial)) or TS=(Cancers, Endometrial)) or TS=(Endometrial Cancers)) AND (((((((((((((((((((((((((((((((((((((((((((((((((((((((TS=(Checkpoint Inhibitors, Immune)) or TS=(Immune Checkpoint Blockers)) or TS=(Checkpoint Blockers, Immune)) or TS=(Immune Checkpoint Inhibitor)) or TS=(Checkpoint Inhibitor, Immune)) or TS=(CTLA-4 Inhibitors)) or TS=(CTLA 4 Inhibitors)) or TS=(Cytotoxic T-Lymphocyte-Associated Protein 4 Inhibitors)) or TS=(Cytotoxic T Lymphocyte Associated Protein 4 Inhibitors)) or TS=(Cytotoxic T-Lymphocyte-Associated Protein 4 Inhibitor)) or TS=(Cytotoxic T Lymphocyte Associated Protein 4 Inhibitor)) or TS=(CTLA-4 Inhibitor)) or TS=(CTLA 4 Inhibitor)) or TS=(PD-1 Inhibitors)) or TS=(PD 1 Inhibitors)) or TS=(Programmed Cell Death Protein 1 Inhibitor)) or TS=(Programmed Cell Death Protein 1 Inhibitors)) or TS=(PD-1 Inhibitor)) or TS=(Inhibitor, PD-1)) or TS=(PD 1 Inhibitor)) or TS=(Immune Checkpoint Blockade)) or TS=(Checkpoint Blockade, Immune)) or TS=(Immune Checkpoint Inhibition)) or TS=(Checkpoint Inhibition, Immune)) or TS=(PD-L1 Inhibitors)) or TS=(PD L1 Inhibitors)) or TS=(Programmed Death-Ligand 1 Inhibitors)) or TS=(Programmed Death Ligand 1 Inhibitors)) or TS=(PD-L1 Inhibitor)) or TS=(PD L1 Inhibitor)) or TS=(PD-1-PD-L1 Blockade)) or TS=(Blockade, PD-1-PD-L1)) or TS=(PD 1 PD L1 Blockade)) or TS=(pembrolizumab)) or TS=(nivolumab)) or TS=(dostarlimab)) or TS=(cemiplimab)) or TS=(retifanlimab)) or TS=(tislelizumab)) or TS=(toripalimab)) or TS=(sintilimab)) or TS=(camrelizumab)) or TS=(cindilimab)) or TS=(penpulimab)) or TS=(serplulimab)) or TS=(pucotenlimab)) or TS=(avelumab)) or TS=(atezolizumab)) or TS=(durvalumab)) or TS=(cosibelimab)) or TS=(sugemalimab)) or TS=(envafolimab)) or TS=(adebrelimab)) or TS=(ipilimumab)) or TS=(tremelimumab)) | 2015-01-01  ——  2024-12-31 | 1,140 |
| Query #2 | #1 and Article or Review Article (Document Types) | 2015-01-01  ——  2024-12-31 | 860 |
| Query #3 | #1 and Article or Review Article (Document Types) and English (Languages) | 2015-01-01  ——  2024-12-31 | 845 |
| Query #4 | Excluded 16 articles not related to the study of immune checkpoint inhibitors in endometrial cancer. | 2015-01-01  ——  2024-12-31 | 829 |
