# Supplementary material for: A systematic review of immune checkpoint inhibitors in endometrial cancer
Source: Front Oncol. 2026 Jun 8;16:1776831. doi: 10.3389/fonc.2026.1776831 (PMC13284135; doi:10.3389/fonc.2026.1776831)
Supplement: Supplementary file 2 [file Table2.docx]

**Supplementary File 2.Search strategy of PubMed Database**

| **Search strategy of PubMed Database** | Term | Date | Retrieval results |
| --- | --- | --- | --- |
| Query #1 | (((((((((((((((((((((((((((((((((((((((((Endometrial Neoplasm[Title/Abstract])) or (Neoplasm, Endometrial[Title/Abstract])) or (Neoplasms, Endometrial[Title/Abstract])) or (Endometrial Carcinoma[Title/Abstract])) or (Carcinoma, Endometrial[Title/Abstract])) or (Carcinomas, Endometrial[Title/Abstract])) or (Endometrial Carcinomas[Title/Abstract])) or (Cancer of Endometrium[Title/Abstract])) or (Endometrium Cancers[Title/Abstract])) or (Endometrium Cancer[Title/Abstract])) or (Cancer, Endometrium[Title/Abstract])) or (Cancers, Endometrium[Title/Abstract])) or (Cancer of the Endometrium[Title/Abstract])) or (Carcinoma of Endometrium[Title/Abstract])) or (Endometrium Carcinoma[Title/Abstract])) or (Endometrium Carcinomas[Title/Abstract])) or (Endometrial Cancer[Title/Abstract])) or (Cancer, Endometrial[Title/Abstract])) or (Cancers, Endometrial[Title/Abstract])) or (Endometrial Cancers[Title/Abstract])) or (Endometrial Neoplasm[Text Word])) or (Neoplasm, Endometrial[Text Word])) or (Neoplasms, Endometrial[Text Word])) or (Endometrial Carcinoma[Text Word])) or (Carcinoma, Endometrial[Text Word])) or (Carcinomas, Endometrial[Text Word])) or (Endometrial Carcinomas[Text Word])) or (Cancer of Endometrium[Text Word])) or (Endometrium Cancers[Text Word])) or (Endometrium Cancer[Text Word])) or (Cancer, Endometrium[Text Word])) or (Cancers, Endometrium[Text Word])) or (Cancer of the Endometrium[Text Word])) or (Carcinoma of Endometrium[Text Word])) or (Endometrium Carcinoma[Text Word])) or (Endometrium Carcinomas[Text Word])) or (Endometrial Cancer[Text Word])) or (Cancer, Endometrial[Text Word])) or (Cancers, Endometrial[Text Word])) or (Endometrial Cancers[Text Word])) AND (((((((((((((((((((((((((((((((((((((((((((((((((((((((((((((((((((((((((((((((((((((((((((((((((((((((((((((((Checkpoint Inhibitors, Immune[Title/Abstract])) or (Immune Checkpoint Blockers[Title/Abstract])) or (Checkpoint Blockers, Immune[Title/Abstract])) or (Immune Checkpoint Inhibitor[Title/Abstract])) or (Checkpoint Inhibitor, Immune[Title/Abstract])) or (CTLA-4 Inhibitors[Title/Abstract])) or (CTLA 4 Inhibitors[Title/Abstract])) or (Cytotoxic T-Lymphocyte-Associated Protein 4 Inhibitors[Title/Abstract])) or (Cytotoxic T Lymphocyte Associated Protein 4 Inhibitors[Title/Abstract])) or (Cytotoxic T-Lymphocyte-Associated Protein 4 Inhibitor[Title/Abstract])) or (Cytotoxic T Lymphocyte Associated Protein 4 Inhibitor[Title/Abstract])) or (CTLA-4 Inhibitor[Title/Abstract])) or (CTLA 4 Inhibitor[Title/Abstract])) or (PD-1 Inhibitors[Title/Abstract])) or (PD 1 Inhibitors[Title/Abstract])) or (Programmed Cell Death Protein 1 Inhibitor[Title/Abstract])) or (Programmed Cell Death Protein 1 Inhibitors[Title/Abstract])) or (PD-1 Inhibitor[Title/Abstract])) or (Inhibitor, PD-1[Title/Abstract])) or (PD 1 Inhibitor[Title/Abstract])) or (Immune Checkpoint Blockade[Title/Abstract])) or (Checkpoint Blockade, Immune[Title/Abstract])) or (Immune Checkpoint Inhibition[Title/Abstract])) or (Checkpoint Inhibition, Immune[Title/Abstract])) or (PD-L1 Inhibitors[Title/Abstract])) or (PD L1 Inhibitors[Title/Abstract])) or (Programmed Death-Ligand 1 Inhibitors[Title/Abstract])) or (Programmed Death Ligand 1 Inhibitors[Title/Abstract])) or (PD-L1 Inhibitor[Title/Abstract])) or (PD L1 Inhibitor[Title/Abstract])) or (PD-1-PD-L1 Blockade[Title/Abstract])) or (Blockade, PD-1-PD-L1[Title/Abstract])) or (PD 1 PD L1 Blockade[Title/Abstract])) or (pembrolizumab[Title/Abstract])) or (nivolumab[Title/Abstract])) or (dostarlimab[Title/Abstract])) or (cemiplimab[Title/Abstract])) or (retifanlimab[Title/Abstract])) or (tislelizumab[Title/Abstract])) or (toripalimab[Title/Abstract])) or (sintilimab[Title/Abstract])) or (camrelizumab[Title/Abstract])) or (cindilimab[Title/Abstract])) or (penpulimab[Title/Abstract])) or (serplulimab[Title/Abstract])) or (pucotenlimab[Title/Abstract])) or (avelumab[Title/Abstract])) or (atezolizumab[Title/Abstract])) or (durvalumab[Title/Abstract])) or (cosibelimab[Title/Abstract])) or (sugemalimab[Title/Abstract])) or (envafolimab[Title/Abstract])) or (adebrelimab[Title/Abstract])) or (ipilimumab[Title/Abstract])) or (tremelimumab[Title/Abstract])) or (Checkpoint Inhibitors, Immune[Text Word])) or (Immune Checkpoint Blockers[Text Word])) or (Checkpoint Blockers, Immune[Text Word])) or (Immune Checkpoint Inhibitor[Text Word])) or (Checkpoint Inhibitor, Immune[Text Word])) or (CTLA-4 Inhibitors[Text Word])) or (CTLA 4 Inhibitors[Text Word])) or (Cytotoxic T-Lymphocyte-Associated Protein 4 Inhibitors[Text Word])) or (Cytotoxic T Lymphocyte Associated Protein 4 Inhibitors[Text Word])) or (Cytotoxic T-Lymphocyte-Associated Protein 4 Inhibitor[Text Word])) or (Cytotoxic T Lymphocyte Associated Protein 4 Inhibitor[Text Word])) or (CTLA-4 Inhibitor[Text Word])) or (CTLA 4 Inhibitor[Text Word])) or (PD-1 Inhibitors[Text Word])) or (PD 1 Inhibitors[Text Word])) or (Programmed Cell Death Protein 1 Inhibitor[Text Word])) or (Programmed Cell Death Protein 1 Inhibitors[Text Word])) or (PD-1 Inhibitor[Text Word])) or (Inhibitor, PD-1[Text Word])) or (PD 1 Inhibitor[Text Word])) or (Immune Checkpoint Blockade[Text Word])) or (Checkpoint Blockade, Immune[Text Word])) or (Immune Checkpoint Inhibition[Text Word])) or (Checkpoint Inhibition, Immune[Text Word])) or (PD-L1 Inhibitors[Text Word])) or (PD L1 Inhibitors[Text Word])) or (Programmed Death-Ligand 1 Inhibitors[Text Word])) or (Programmed Death Ligand 1 Inhibitors[Text Word])) or (PD-L1 Inhibitor[Text Word])) or (PD L1 Inhibitor[Text Word])) or (PD-1-PD-L1 Blockade[Text Word])) or (Blockade, PD-1-PD-L1[Text Word])) or (PD 1 PD L1 Blockade[Text Word])) or (pembrolizumab[Text Word])) or (nivolumab[Text Word])) or (dostarlimab[Text Word])) or (cemiplimab[Text Word])) or (retifanlimab[Text Word])) or (tislelizumab[Text Word])) or (toripalimab[Text Word])) or (sintilimab[Text Word])) or (camrelizumab[Text Word])) or (cindilimab[Text Word])) or (penpulimab[Text Word])) or (serplulimab[Text Word])) or (pucotenlimab[Text Word])) or (avelumab[Text Word])) or (atezolizumab[Text Word])) or (durvalumab[Text Word])) or (cosibelimab[Text Word])) or (sugemalimab[Text Word])) or (envafolimab[Text Word])) or (adebrelimab[Text Word])) or (ipilimumab[Text Word])) or (tremelimumab[Text Word])) | 2015-01-01  ——  2024-12-31 | 497 |
| Query #2 | #1 and Case Reports or Clinical Study or Clinical Trial or Meta-Analysis or Randomized Controlled Trial or Review or Systematic Review (Document Types) | 2015-01-01  ——  2024-12-31 | 282 |
| Query #3 | #1 and Case Reports or Clinical Study or Clinical Trial or Meta-Analysis or Randomized Controlled Trial or Review or Systematic Review (Document Types) and English (Languages) | 2015-01-01  ——  2024-12-31 | 279 |
